# Assessment of cognitive performance and fatigability in elite athletes: Short and portable protocols for field monitoring under hypoxia

**DOI:** 10.1371/journal.pone.0353673

**Published:** 2026-07-10

**Authors:** Giorgio Varesco, Nicolas Bourrel, François Bieuzen, Guido Simonelli

**Affiliations:** 1 National Institute of Sport of Québec, Québec, Canada; 2 Center for Advanced Research in Sleep Medicine, Hôpital du Sacré-Coeur de Montréal, CIUSSS du Nord de l’Île-de- Montréal, Québec, Canada; 3 Departement of Medicine, Université de Montréal, Québec, Canada; 4 Departement of Neuroscience, Université de Montréal, Québec, Canada; Portugal Football School, Portuguese Football Federation, PORTUGAL

## Abstract

**Purpose:**

This study aimed to evaluate whether short (~15 min) cognitive protocols can detect fatigability in normoxia versus normobaric hypoxia in elite youth athletes, using tasks directly transferable to the field.

**Methods:**

Elite youth mogul skiers (18 ± 2 y) completed two studies. In Study I, 17 athletes (7 women) performed a cognitive test after a repeated tuck-jump protocol designed to mimic mid-training physical demands under two conditions in fixed order: sham normoxia (simulated 200 m) and normobaric hypoxia (3500 m, blood saturation ~88%). In Study II, 16 athletes (6 women) performed the same cognitive test without the jump task, with conditions randomized. In both studies, response time and accuracy during a 10-min color Multisource Interference Task (cMSIT) were used to assess cognitive performance, and a 3-min Psychomotor Vigilance Task (PVT) was administered before and after the cMSIT to quantify fatigability. Cognitive tests were performed on portable tablet devices.

**Results:**

After adjusting for baseline cognitive performance, cMSIT response time was greater in hypoxia than in normoxia in both Study I (10 ± 2%, p = 0.004) and Study II (4 ± 1%, p < 0.001). Accuracy remained high and similar across conditions in both studies (Study I: 96 ± 3%; Study II: 96 ± 0.5%; p > 0.09). In Study I, PVT response time increased only in hypoxia, from 363 ± 74 ms to 375 ± 89 ms (p = 0.021), and lapses increased from 1 ± 0.5 to 3 ± 2.5 (p < 0.001), whereas lapses remained unchanged in normoxia and in Study II (p > 0.82). In Study I, jump task performance showed a condition×set interaction (p = 0.048), with lower performance in the third set in hypoxia (19806 ± 2464 N·s) than in normoxia (20130 ± 3016 N·s; p = 0.012).

**Conclusions:**

In elite youth athletes, hypoxemia reduces cognitive performance and could lead to increased fatigability. Importantly, hypoxia-related impairments are detectable with short portable cognitive assessments, which are promising tools for field monitoring, particularly when tasks combines cognitive and physical demands.

## Introduction

Several occupations and athletic disciplines necessitate performance at high altitudes (2500–3500 m [1]). This is particularly relevant for people involved in winter sports and the alpine tourism industry, as ongoing climate change progressively reduces snow cover at lower elevations [2]. These individuals are frequently subjected to intermittent hypoxic exposure, training, competing or working at high altitudes while residing or sleeping at lower elevations. Due to its crucial importance in injury prevention or performance in tasks with high cognitive demands, the impact of hypoxemia on cognitive function is receiving increasing attention. Because tasks that closely mirror the cognitive demands of sport or work are often impractical at altitude, researchers typically use standardized computer-based behavioral tasks to balance feasibility with contextual specificity [3,4]. Studies evaluating the effect of short-term hypoxia exposure (e.g., < 2h) on cognitive function report conflicting results and remain mainly confined to long, laboratory-based protocols [5]. Furthermore, amidst the focus on cognitive executive functions at altitude, monitoring of fatigability (or endurance) has been less investigated.

In the present work, fatigability refers to the rate of decrement in an objective performance criterion at a given task [6]. Fatigability is influenced by task characteristics, individual states and traits, and environmental conditions, and it often co-occurs with fatigue. We distinguish fatigability from fatigue, defined as the subjectively perceived feeling of lack of energy, which is not necessarily accompanied by a decrease in objective performance [7]. Fatigability can be quantified continuously or as the difference in performance at a physical or cognitive test performed before, during, and after a task [6,8]. The more the performance test can measure the maximal capacity of a system (e.g., maximal force for the neuromuscular system), the more sensitive it is to detect performance impairments. Such impairments might be missed if testing is done at a submaximal level, where the sensitivity is lower.

While this is obvious for neuromuscular testing [8], translating a similar framework to the cognitive domain is more complex.

In the literature, computer-based tasks involving core executive functions are often used to assess cognitive fatigability under sustained task demands [9,10]. Inhibitory control tasks are frequently selected because they are relevant to sport settings, sensitive to fatigue [4,11] and involve processes that overlap with other executive functions [10]. Sustained attention is another key marker of cognitive performance and fatigability [12,13]. It is often quantified using the psychomotor vigilance task (PVT), a validated reaction-time task [14–16]. Due to its short duration (i.e., 3 min for the short version), PVT can be performed before and after the fatiguing task, providing a practical way to further characterize cognitive fatigability [15].

Under hypoxia, studies investigating fatigability report inconsistent findings: for instance, De Watcher et al. [13] compared performance on a 10-minute PVT and tasks involving working memory and visual tracking, performed before and after 60 min of either a Stroop task or neutral video in normoxia vs. hypoxia (13.5% FiO_2_, corresponding to ~3500 m). They found no effect of hypoxemia on fatigability both in terms of PVT and Stroop task performance. O’Keeffe et al. [17] compared the performance at a Tower of Hanoi test and at physical tests (self-paced arm-cycling and maximal isometric contractions) performed in normoxia vs. hypoxia (≥3500 m) after either a 16-minute TloadDback task or a neutral video. Hypoxemia influenced the performance at the physical tasks, but no increase in cognitive fatigability was detected, which the authors attributed to the fact that the TloadDback was always performed in normoxia and to high interindividual variability underpowering the results [17]. Conversely, Ochi et al. [11] found increased cognitive fatigability in hypoxia (~3500 m) vs. normoxia measured as the performance decline at a 6.5-minute Stroop task performed before and after 10 min of moderate exercise. This decline was accompanied by an increased activity of areas involved in inhibitory control, such as the prefrontal cortex (PFC) [11].

While valid, the field applicability of current protocols is limited by their long duration and laboratory format, highlighting the need for short, validated tests in hypoxic conditions. Moreover, interindividual variability in fitness and cognitive profiles could act as an important confounder, possibly masking hypoxia effects. Currently, there is a need for improvements in transferability of these measures to the field (e.g., [18]). Using short, portable tasks would allow for valid and repeated assessments of cognitive fatigability in training conditions, addressing the growing need for appropriate daily monitoring in the field.

Thus, the current study aims to evaluate cognitive fatigability in normoxia vs. hypoxia in late-adolescent elite Canadian ski-mogul athletes, a lowlander population of elite youth athletes that often trains and competes at altitude, by mimicking a mid-training situation (e.g., performing a physical task before cognitive testing) [19,20]. We wanted to evaluate the ability of short (~15 min) protocols to detect fatigability in this population, addressing current gaps in field-deployable measures by adopting tasks with limited margin for strategy optimization (learning effect), culturally neutral, and performed using an open-source touchscreen app we developed. Because inter-trial recovery was not allowed during the cognitive task, we hypothesized the presence of objective fatigability in both hypoxia and normoxia, unlike De Watcher et al. [13]. We further hypothesized that hypoxia would exacerbate fatigability, both after a physical task, as in Ochi et al. [11], and in the absence of prior physical exercise.

## Methods

### Study design overview

To address our research question, we tested the Quebec’s Freestyle Moguls team in 2025 (Study I) and 2026 (Study II). Study I examined whether a short protocol detects fatigability changes in normoxia versus hypoxia simulating a mid-training situation blinding participants of the condition (sham vs. true hypoxia), which necessitated fixed-order sessions. Study II addressed this limitation by testing the team one year later to standardize training load and season period, using a crossover design with two randomized groups balanced for age and sex. One group performed cognitive testing in hypoxia during the first session and the other during the second, with no jump task to isolate effect of hypoxia on cognitive performance.

### Participants

For the first study, seventeen youth elite athletes (7 women, age: 18 ± 2 y; body mass: 69 ± 10 kg; height: 175 ± 10 cm) of the 2025 team were fully informed on the testing procedures, but naïve to the goal of the study. Athletes had 9 ± 2 yr of competition experience, including 2 ± 1 yr at the elite level. Athletes underwent a series of questionnaires to exclude the presence of sleep disturbances (Pittsburgh Sleep Quality Index: 5 ± 2; Insomnia Severity Index: 6 ± 4), excessive daytime fatigue (Brugmann scale: mental item = 3 ± 1, physical item = 3 ± 2), and sleepiness (Epworth scale: 7 ± 3). Chronotype was intermediate (Caen Chronotype Questionnaire v2: 58 ± 16%).

The composition of the 2026 team roster changed: in Study II we tested 16 athletes (6 women, 18 ± 2 y, 68 ± 13 kg, 172 ± 12 cm) with 8 ± 2 y of competition experience (2 ± 1 y at elite level). The group showed no presence of sleep disturbances (Pittsburgh Sleep Quality Index: 4 ± 2; Insomnia Severity Index: 6 ± 4), excessive daytime fatigue (Brugmann scale: mental item = 2 ± 1, physical item = 2 ± 1), and sleepiness (Epworth scale: 6 ± 3). Chronotype was morning to intermediate (Caen Chronotype Questionnaire v2: 31 ± 16%). All athletes complied with the inclusion criteria, which consisted of the absence of medical conditions impacting cognitive function and/or sleep, or posing an increased risk of negative outcomes at altitude, such as epilepsy and heart conditions. None of the athletes took part in previous studies evaluating the effects of hypoxia, and none of our participants entered a hypoxic chamber before. Elite youth mogul athletes represent a limited cohort (~300–400 U23 active FIS athletes worldwide). Our convenience sample of ~20 elite athletes represents 5% of the total population. The project procedures adhere to the Declaration of Helsinki (2013), and was approved by the Ethical Research Committee of the Centre Integré Universitaire de Santé et de Services Sociaux du Nord-de-l’Île-de-Montréal (CIUSSS-NIM n. 2024–2718). All athletes provided written consent (co-signed by their legal guardian in case of a minor) to participate in the present study. The recruitment period ran from 01/04/2025 to 30/05/2026.

### Study I: Experimental Procedures

**Fig 1:**
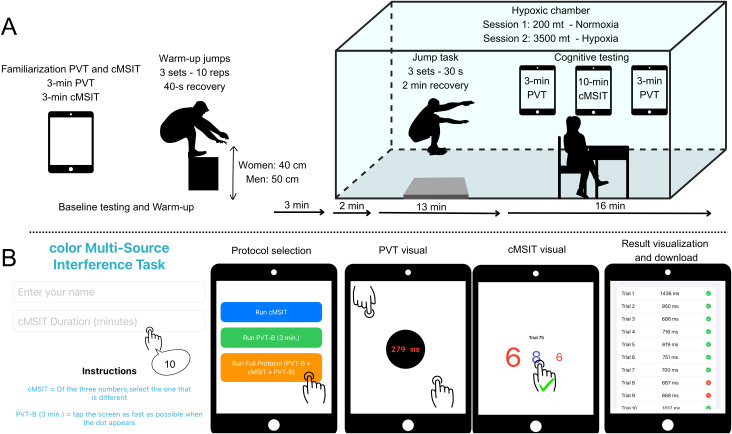
Illustration of the testing procedure for each experimental session (Panel A) and visual of the cMSIT app interface used for the cognitive testing (Panel B). Once the duration of the task (e.g., 10 min) was specified and the appropriate protocol was selected, the task commenced. In the Psychomotor Vigilance Task (PVT), once the visual stimuli and the timer appear, the user can touch any part of the screen to respond. For the color Multi-Source Interference Task, the user needs to touch the correct answer (i.e., the number that is different from the other two). At task completion, results can be visualized and downloaded locally on the device. Note that if the full protocol including a 3 min PVT performed before and after a cMSIT is selected, the results will be presented only after the second PVT, i.e., at the end of the protocol.

After inclusion, the 2025 team visited the laboratory on 2 different occasions, separated by precisely two weeks, and were all tested in the same order between 9:30 AM and 1 PM. These experimental sessions, illustrated in Fig 1A, were positioned at the beginning of the pre-season (May-June 2025). Training load was adjusted in collaboration with the coaches to i) avoid excessive fatigue prior to the experimental session (i.e., no exercise 24h prior to the test) and ii) keep consistent training load across weeks preceding the study. Familiarization and practice trials on a PVT and a color Multi-Source Interference Task (cMSIT; Fig 1B) were conducted at the start of both sessions to address possible novelty or boredom effects from one session to another. Successively, a 3-minute cMSIT and a 3-minute PVT were performed to assess participants cognitive performance at baseline. Then, they performed a physical warm-up, consisting of 3 sets of 10 step-up jumps (40 s recovery) on a cube of 40 cm for women and 50 cm for men. For both sessions, the remaining experimental procedures were performed in a normobaric hypoxic chamber controlled by a validated altitude generator (Everest Summit II, Hypoxico Inc., GER; [21]). In the first and second session, the room was set to simulate an altitude of 200 m (normoxic condition), and 3500 m (hypoxic condition), respectively. The temperature was set to be 21 ± 0.5°C, with 50% of relative humidity. In the hypoxic chamber, participants first rested for two minutes seated, then performed a moguls-specific jump task within 5 minutes after the warm-up. After 5.5 minutes of rest, i.e., once athletes had been in the chamber for 15 min, the cognitive test started. The cognitive testing for fatigability consisted of 3 minutes of PVT performed before and after 10 minutes of cMSIT (Fig 1A). The jump task was placed before cognitive testing to enhance i) the comparability with prior work employing exercise-induced fatigue prior to cognitive testing [13,17,22]), and ii) the ecological validity of the protocol and mimic mid-training cognitive demands during moguls training sessions [19,20]. This reflects real-world training scenarios where a certain amount of physical stress is concomitant with cognitive processing, such as aerial preparation or course navigation, thereby increasing the protocol’s representativeness for assessing cognitive fatigability in applied contexts.

Participants were naïve to the condition and were told that the chamber was operating to simulate 3500 m of altitude in both sessions. The reasons for simulating 200 m in the normoxic condition were to i) better control for environmental conditions and temperature, and ii) make the generators operate (noise) to convince athletes that altitude was simulated in both conditions. A randomized crossover design was not feasible because the altitude simulator system requires 45–120 min to stabilize a chosen simulated altitude. Randomizing the participants to the condition would necessitate waiting ~2 hours between athletes or subgroups to pass from 200 m to 3500 m (or vice versa), making the protocol unfeasible. Testing different athletes on different days was not viable given their constrained training schedules. Due to the use of a sham condition (200 m), participants were not informed of the true altitude exposure until after data collection. All participants were debriefed post-study, and feedback regarding the study procedures and aims was provided.

### Cognitive testing

We designed an app (source code openly available at https://doi.org/10.17605/OSF.IO/D75YU) for iOS devices which included options to perform cMSIT and PVT tests (Fig 1B). The cMSIT stresses the inhibitory control executive function, similarly to the Stroop, Eriksen or Simon tasks and it involves an important activation of the PFC and the dorsal anterior cingulate cortex (dACC; [23,24]). The cMSIT has the additional advantage of being entirely based on numbers, which is preferential in bilingual regions such as Québec (French/English), as the cognitive demands of tasks based on word recognition, such as the Stroop task (commonly used in the literature), vary greatly among individuals with bilingual backgrounds [25]. We previously showed that a 30-min cMSIT consistently induces impairments in youth elite athletes [26]. During the cMSIT, three numbers ranging from 1 to 9 (2° × 1.5° of visual angle separated by 2°; participants were positioned ~30 cm from the screen) were displayed on the screen of a tablet with the set of numbers randomized for each trial. In each set, two of the numbers are identical, while the third is different (e.g., 7-7-1, 2-3-2, 6-6-9). The goal is to select the different number as quickly as possible, ignoring the numerical value, color and size to provide the correct response (Fig 1B). Trials were separated by 0 s. The numbers presented varied also in size and color. Colors were coded to be color-blind friendly (IBM-DLC palette hex: #785ef0; #dc267f). Athletes were informed that they needed to achieve the highest number of correct answers within the 10-minute time. For each trial, we exported response time, accuracy (correct or incorrect response) and the number combination. We then calculated, for each minute of the test, the mean response time, the total number of correct answers and the total number of trials performed.

The PVT was coded to replicate the PVT-192 (Ambulatory Monitoring Inc., Ardsley, NY), which delivers a visual stimulus at intervals varying from 2 to 10 s. Once the visual stimulus appears (a time counter), subjects need to press a button as fast as possible [15,27]. The counter ran for 2.5 s. In our version for touchscreen, the stimuli consisted of a black dot with a red timer, appearing on the blank screen (Fig 1B). Athletes were instructed to tap the screen as fast as possible, and it was possible to touch any part of the screen. Touch screen-based PVT has been shown to be as valid as the original PVT-192 device [28]. Main outcomes for the PVT were the number of lapses and response time. Response times>1s were excluded to avoid excessive skew when calculating response time (49/2429 trials; i.e., 1.96%) [29]. Response times <100 ms were counted as false starts. Thus, PVT response time consisted of responses included between 0.1 and 1 s over the 3-minute PVT. Lapses were initially considered as response times>355 ms and >500 ms [14,15]. However, the > 355 ms threshold was not appropriate as it captured ~50% of responses rather than attentional lapses (see Results). Only a threshold for lapses >500 ms was adopted [14,15,29]. Cognitive fatigability was defined as the decline in PVT performance (slower response times, increased lapses) from pre-to post-cMSIT. The tablet used (iPad 10th generation, 2022, A14 Bionic; iOS 18.7.7) featured a 60 Hz touchscreen polling (~16.7 ms interval) and end-to-end latency of 70–90 ms per benchmarks, which justifies critical re-evaluation of the threshold for lapses that we performed following the rationale presented in Basner et al. [14] original PVT work.

### Jump task

The task consisted of 3 sets of 30 seconds of repeated tuck jumps on forceplates (ForceDecks Lite, Vald Performance, AUS). Sets were separated by 2 minutes of rest, where athletes remained seated on a chair. Similar repeated tuck jumps tasks are routinely adopted by mogul athletes in training to help stabilize the upper part of the body, reducing the center of mass displacement during each jump. Athletes were instructed to perform as many jumps as possible with the maximal jump height to obtain the highest cumulative impulse for each 30 s set. The timing (30 s) was consistent with a typical run on a ski-moguls slope. A timer automatically started when participants initiated the first countermovement, and an audio signal was provided when the 30-s elapsed. Jumps whose concentric phase commenced before the 30 s audio signal were considered valid. Propulsive impulse for each jump was determined as the net force-time integral during the concentric phase, from zero-velocity crossing to takeoff using the Forcedecks software. Impulse for each jump was summed to obtain total impulse for each series. Before entering the chamber, athletes wore a pulse oximeter (WristOx2 3150, Nonin, MN) to keep at rest and during the jump task. Once signal quality was checked, the screen was covered with black tape to prevent feedback to the athlete after entering the chamber. However, heart rate and saturation data resulted mostly unusable once the jump task started, possibly due to the movements of the participants during the jumps, displacing the sensor and the cables. As such, only the oximeter data at rest (before the test) are presented.

### Study II: Reproducibility

The main limitations of Study I were non-randomized sessions and the inability to separate the effect of the jump task on cognitive performance. To address these limitations and improve internal validity, we collected data one year later on the same team during the pre-season period. Athletes were tested 3 weeks apart and assigned to two groups balanced for age and sex but otherwise randomized. Procedures remained similar to Study I (familiarization, baseline testing, and cognitive testing), but one group performed cognitive testing in hypoxia during the first session while the other did so during the second session, in a crossover design. No jump task was performed, isolating hypoxia’s effect on cognitive performance. Athletes were tested in the same order and time. Finally, athletes filled out the NASA-Task Load Index questionnaire (NASA-TLX) [30] to report their perceived workload regarding the cognitive testing, since perceived workload was not assessed in Study I.

### Statistical analysis

Analyses were performed in the R environment (V4.2.3, R Foundation for Statistical Computing, Vienna, AUT). Data are presented as mean±SD unless otherwise specified. For the cMSIT, we evaluated the effect of condition×time (minute on task, 1–10) on the mean response time and accuracy, i.e., the number of correct answers adjusted for the total number of trials. To account for inter-session differences in baseline cognitive performance, we also adjusted cMSIT data for day-specific cognitive performance (i.e., data at the 3-min cMSIT performed before entering the hypoxic chamber were added as a term in the model). For the PVT, we evaluated the effects of condition×time (pre- to post-cMSIT) on lapses and response time. Since PVT analysis consisted of a pre- to post-cMSIT difference, there was no need for baseline adjustment. Regardless, we compared PVT lapses and response time between sessions at baseline to check for differences in sustained attention. For the jump task (Study I only), we evaluated total impulse adjusted for body mass as an index of performance at the test. Previous studies have shown that body mass (continuous variable), rather than binary sex, provides superior prediction of jump performance outcomes [31] (see Supporting Information S2 File for demonstration and data divided by sex). For cognitive testing, the sex factor was removed from the models as it did not affect the results (all p > 0.07). Similarly, we adjusted our estimate for hour of testing, but it was removed from the model if it had no effect (Occam razor principle). Hour of testing was kept only for cMSIT response time in Study I (p < 0.02). For Study II, we evaluated the difference across conditions for all items of the NASA-TLX, and if any of the items were correlated with cMSIT response time (Pearson correlation coefficient).

For all analyses, we implemented linear mixed-effects models (LMM; lme4 package), with a random intercept for participants to account for individual variability, unless otherwise specified. Estimates were calculated using the least-squares means method. For all models, the empirical test of the model assumptions was performed via model residuals graphical analysis of the Q-Q plots. Analysis of deviance (Type III Wald χ^2^ tests) was used to evaluate the global effects of sleep conditions, with p-values obtained from F-tests using Satterthwaite’s degrees of freedom method (lmerTest package). Effect sizes (partial eta squared, η^2^p; effectsize package) were interpreted as small (η^2^p>0.01), medium (η^2^p>0.06), and large (η^2^p>0.14). Post-hoc analyses using Tukey’s HSD test (emmeans package) were conducted to explore pairwise differences when significant main effects or interactions were observed. For all statistical tests, α was set at 0.05. The complete code used for analyses is consultable at https://doi.org/10.17605/OSF.IO/D75YU.

## Results

In 2025, among the 17 athletes invited, 1 athlete did not participate in the normoxia session and 2 in the hypoxia session due to influenza (different individuals). In 2026, all 16 participants underwent both sessions. SpO₂ was 98 ± 1% in normoxia and 88 ± 2% in hypoxia.

### Study I

#### Cognitive performance at baseline.

**Fig 2:**
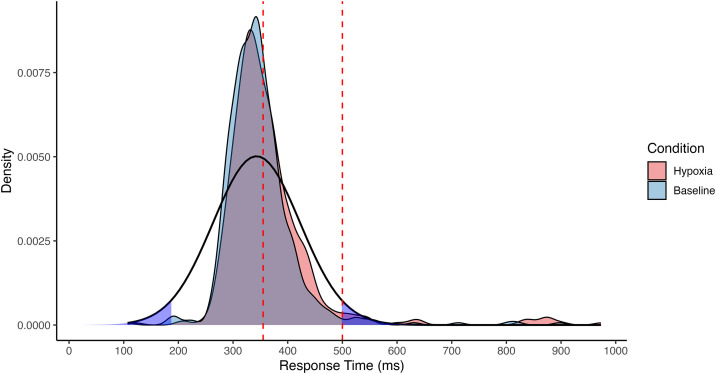
Density distribution of response times from the Psychomotor Vigilance Test (PVT) at baseline for the 2 experimental sessions. Of note, these data were obtained before performing any fatiguing task and before entering the hypoxic chamber. Data distribution is overlaid with a normal distribution curve centered at the data’s median (due to skewed distribution). Vertical red lines mark the conventional PVT lapse thresholds of 355 ms and 500 ms, while dark blue shading highlights the theoretical 2.5% tails (α = 0.05, two-tailed) of the fitted normal distribution.

Before entering the hypoxic chamber, no difference in lapses between sessions was found for both >355 ms (16 ± 10 lapses; η^2^p=0.10; p = 0.32) and >500 ms (2 ± 2 lapses; η^2^p=0.05; p = 0.26). The 355 ms threshold bisected the response time distribution near its median, capturing approximately 50% of responses rather than attentional lapses (Fig 2). In contrast, the 500 ms threshold was closely aligned with the upper 2.5% tail when fitting data to a normal distribution (Fig 2). For mean response time at the PVT, the Q-Q plot of the LMM residuals showed the presence of some long response times that influenced model fit. Thus, a robust linear mixed model (robustlmm package) was implemented to evaluate differences between session days, showing similar results (Fig 2; p = 0.49).

**Fig 3:**
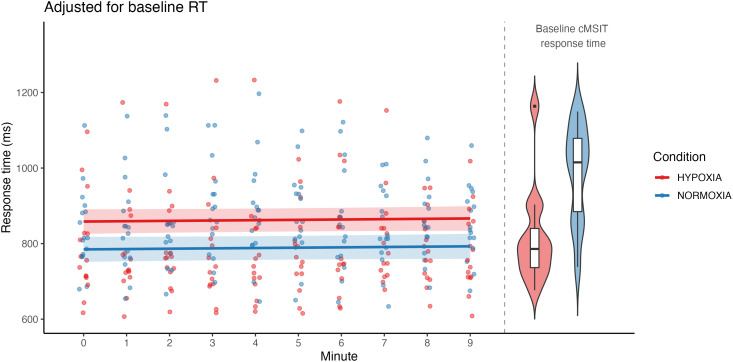
Evolution of response time at the color multisource interference task (cMSIT) across conditions in Study I. Dots represent raw data (unadjusted). Lines and shades represent model prediction ± SE of data adjusted for baseline performance. Baseline data are presented on the right (violin and boxplot) show the average response time at the 3-min cMSIT performed at the beginning of each experimental session.

Baseline cMSIT mean response time was longer in normoxia compared to hypoxia [I ± SE = 822 ± 19 ms; normoxia (β ± SE)=192 ± 26 ms; η^2^p=0.85; p < 0.001; Fig 3]. No difference in cMSIT accuracy was found between conditions at baseline (97 ± 2%; η^2^p=0.11; p = 0.18).

#### Cognitive performance and fatigability.

Mean response time at the cMSIT did not show a condition×time interaction (η^2^p<0.001; p = 0.99) and did not change across the task (time effect: η^2^p=0.001; p = 0.53), while a condition effect was observed (η^2^p=0.19; p < 0.001, Fig 3). Unadjusted cMSIT performance was ~ 7% better in hypoxia than in normoxia [I ± SE = 732 ± 37 ms; normoxia (β ± SE)=58 ± 7 ms; Fig 3]. When adjusting for baseline, performance was ~ 10% worse in hypoxia (β ± SE: −73 ± 17 ms; η^2^p=0.21; p < 0.001, Fig 3). Specific properties of the cMSIT, such as the effect of error and trial combination on response time, partially explain the absence of time effect are presented in Supporting Information S1 File.

Accuracy showed no condition×time interaction (η^2^p=0.01; p = 0.08). These results remained consistent when adjusting for baseline (η^2^p<0.001; p = 0.98). Accuracy at the cMSIT was high (96 ± 3%), and not different across conditions (η^2^p<0.001; p = 0.86). A time effect (η^2^p=0.04; p < 0.001) indicated a decrease of 0.14 ± 0.04% (β ± SE) in accuracy per minute in both conditions, dropping from 72 ± 2 correct answers in the first minute to 70 ± 1 (β ± SE) in the last.

When evaluating fatigability, PVT number of lapses (response time >500ms) showed a right-skewed data distribution. Using a Poisson distribution in a generalized linear mixed model, we found a condition×time interaction (GLMM p-value = 0.009). The model suggests a significant increase in the total number of lapses only for hypoxia (p < 0.001) but not for normoxia (p = 0.82; Table 1).

**Table 1:** Psychomotor vigilance task performance data across time and conditions.

Time	Condition	Lapses (n)^a^	Response time (ms)
Pre	Hypoxia	1 ± 0.5	363 ± 74
	Normoxia	1.5 ± 4.25	365 ± 83
Post	Hypoxia	3 ± 2.5**	375 ± 89*
	Normoxia	1.5 ± 2.5	365 ± 91

Notes: Response time data are expressed as mean±SD. Pre and Post refer to before and after the 10 min color-Multisource Interference Task, respectively. ^a^ = differences estimated using generalized linear mixed models, data presented are median±interquartile range. * = difference from pre (same condition). *: p < 0.05; ***: p < 0.001.

PVT response time showed a condition×time interaction (η^2^p=0.02; p = 0.043). At pre-cMSIT, PVT response time was not different across conditions (p = 0.99) while response time was greater post-cMSIT in hypoxia (p = 0.021; Table 1). From the density distribution and Q-Q plot analyses of residuals, some long response times (e.g., > 500ms) might bias these results. However, results were confirmed by robust linear mixed models (condition×time interaction: p = 0.003).

#### Jump Task.

One athlete was excluded from performing the jump task in both sessions due to knee pain, and another athlete did not perform it in normoxia due to back pain. Fourteen athletes performed both jump tasks.

A condition×set interaction was found (η^2^p=0.10; p = 0.048). Post-hoc analysis revealed no difference between conditions at the first [hypoxia: 19609 ± 2664 N·s; normoxia: 19817 ± 3109 N·s; diff = −319 (95%CI = −882; 245); p = 0.26] and second set [hypoxia: 19535 ± 2607 N·s; normoxia: 19182 ± 2608 N·s; diff = 237 (95%CI = −326; 801); p = 0.40], while at the third set total impulse was lower in hypoxia (19806 ± 2464 N·s) than normoxia [20130 ± 3016 N·s; diff = −766 (95%CI = −1359; −174); p = 0.012].

### Study II

No difference at baseline between conditions was found for PVT response time (343 ± 22 ms, η^2^p<0.001; p = 0.97) and lapses (median±IQR: 1 ± 1; η^2^p=0.003; p = 0.77; 500 ms threshold). Response time at the cMSIT was similar across conditions at baseline (818 ± 111 ms, η^2^p=0.019; p = 0.86).

**Fig 4:**
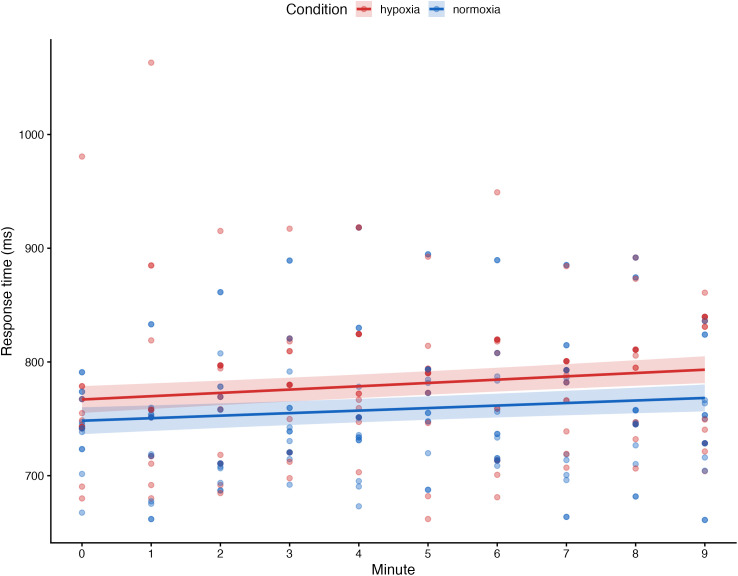
Evolution of response time at the color multisource interference task (cMSIT) across conditions in Study II. Dots represent raw data. Lines and shades represent model prediction±SE of data.

cMSIT performance was lower in hypoxia than in normoxia in both baseline-adjusted (27 ± 5 ms LMM estimated difference, η^2^p=0.08; p < 0.001) and unadjusted models (22 ± 5 ms LMM estimated difference, η^2^p=0.05; p < 0.001; Fig 4). Furthermore, performance decreased with time (−3 ± 1 ms/min, η^2^p=0.03; p = 0.007) in both conditions, with no condition×time interaction (all p > 0.64; Fig 4). Accuracy was high and did not change between conditions (adjusted: 92 ± 2%; η^2^p=0.005; p = 0.22; unadjusted: 96 ± 0.5%; η^2^p=0.009; p = 0.09) or across the task (η^2^p=0.02; p = 0.43).

Finally, performance at the PVT did not change from pre- to post-cMSIT in both conditions in terms of response time (346 ± 17 ms; condition×time: η^2^p<0.001; p = 0.1) and lapses (median±IQR: 1 ± 3ms; condition×time: η^2^p<0.001; p = 0.93). At the NASA-TLX, no item nor weighted score differed across conditions (all p > 0.17). Mental demand was high (54 ± 22 au) and physical demand low (7 ± 9 au). Perceived performance was also high (56 ± 15 au) together with effort (53 ± 15 au), while lower scores were observed for temporal demand (37 ± 22 au) and frustration (40 ± 25 au). Overall weighted score was 37 ± 9 au. None of these items was correlated with cMSIT response time (all p > 0.40).

## Discussion

The main goal of this study was to evaluate cognitive fatigability of elite Canadian youth ski mogul athletes in normoxia vs. hypoxia at a simulated altitude of 3500 m, and to discuss methodological characteristics of the short evaluation protocol used. Our data suggest that performing cognitively demanding tasks at high altitude decreases performance and increases fatigability, which supports our hypothesis. However, contrary to our hypothesis, fatigability was not consistently detected in normoxia using our short protocol.

### Cognitive fatigability under hypoxia

We consistently observed lower performance at the cMSIT under hypoxia vs. normoxia, in line with previous data showing an increase in strain on brain regions like the PFC and dACC with hypoxemia [22,32,33]. Interestingly, with prior physical exercise we also observed i) no change in performance across the task, and ii) a pre- to post-PVT impairment in hypoxia only. In Study I, the presence of a condition effect in cMSIT performance might be interpreted as differences in effort put into the task between sessions, however, a time×condition interaction for cMSIT could be expected in this case. Study II confirmed these results in a different context: without prior exercise, a decline in cMSIT performance was observed with time, without difference across conditions nor changes in PVT performance from pre- to post-cMSIT.

Physical exercise prior to the cognitive task likely altered arousal and effort-regulation processes, thereby modulating performance (pacing) strategy for the cognitive task, while the absence of prior exercise may have reduced this regulation process or arousal, allowing greater time-dependent decline in cMSIT performance [34,35].

Arousal induced by the jump task might compensate for cognitive fatigability, at least in normoxia, while the combination of different stressors (e.g., physical demands, mental demands and hypoxia) produced observable decline in PVT performance. These results support a fatigue-based neurocognitive perspective of executive functioning, proposing that top-down (effort) and bottom-up processes (hypoxemia) act in parallel with arousing mechanisms impacting performance outcomes [36]. Regarding the jump task, it is well established that physical performance deteriorates with hypoxemia: although the intermittent and high-intensity nature of the jump task engages mainly anaerobic energy pathways, which are less impacted by hypoxia (e.g., [37,38]), reliance on aerobic energetic pathways increases with exercise duration and is involved during recovery [39].

These results also suggest that time on task was too short to induce large impairments in our experimental conditions, despite the high cognitive resources mobilized in both normoxia and hypoxia, as shown by the results of the NASA-TLX questionnaire. This is not surprising as athletes usually present lower fatigability compared to non-athletes [40], and longer task durations might be needed. For instance, in a previous study we observed that a 30-minute cMSIT was sufficient to consistently induce impairments in athletes’s PVT performance [26]. Our results underline the need to test for different outcomes that might impact fatigability and performance in real-world scenarios, such as inhibitory control and sustained attention, which both have meaningful implications for athletes in decision-making, technical execution, and safety.

Considering our results, ignoring the effect of hypoxia when planning training at altitude could result in increased fatigability, which might influence negatively the risk of technical errors and thus injuries, especially across multiple days of training.

### Methodological characteristics of the cognitive tasks

Monitoring fatigability using short protocols on mobile devices could be a feasible procedure to implement in the field. To this end, we also evaluated the properties of the touchscreen-based tasks used in the current study. We reported similar response times to other studies validating smartphone-based PVTs (e.g., [28,29]). However, based on our data, a few methodological considerations regarding touchscreen-based PVT can be made for lapses. We observed that the 355 ms threshold, commonly used for the 3-minute PVT to increase lapse sensitivity [14], was inappropriate. In contrast, the 500 ms threshold, i.e., the standard for the 5 and 10-minute PVT [14], was closely aligned with the upper 2.5% tail of response time distribution. In fact, we used a similar rationale to the original work of Basner et al. [14]. Following results from this study and the literature [14,28,29] we propose that the threshold for lapses should be chosen differently according to the device.

Some considerations can also be made for the cMSIT. With a fixed time and zero lag between trials, the task was designed to obtain the maximal number of correct responses in a fixed time (10 min). It was expected to observe a strong and quadratic curvilinear association between the total number of trials and mean response time (Total trials = time on task×response time^-1^). Considering this relationship and the high accuracy observed, we conclude that mean response time and accuracy were appropriate outcomes. Accuracy on the cMSIT could be higher in touchscreen-based tasks than in keyboard-based tasks due to the advantage of directly touching the target response on screen, which reduces oculomotor demands and cognitive load per trial. Previous studies mitigated such effects by limiting numerical variance to 1–3 [23,24]. Here, we used a 9-number version, which likely increased task difficulty and counterbalanced this advantage, as evidenced by response times and accuracy comparable to prior reports in young individuals [41]. Furthermore, we observed that the combination pattern influenced response time, thus ensuring proper randomization of combinations is essential when comparing conditions across sessions or individuals.

Finally, previous literature evaluating cognitive fatigability under hypoxia produced contrasting results, with methodological differences and interindividual variability often mentioned as a limitation [11,13,17,22]. For instance, for a similar FiO_2_ (13.5%; 3500 mt declared), some studies reported a blood saturation of ~92% [13], while others of ~86% [22], which likely influenced fatigability. We reported a blood oxygen saturation of ~88%, which is in line with previous data in normobaric hypoxia when simulating 3500 m [42]. It is thus essential in future studies to report blood oxygen saturation to better characterize its effects on fatigability.

### Limitations and future perspectives

We were aware of the possible learning, fatigue, or expectation effects before the study and adopted all available procedures to address this limitation, such as performing an extensive familiarisation in both sessions, using tasks with limited learning effect and controlling for baseline cognitive performance. However, the potential impact of these limitations on the internal validity of the findings cannot be completely ruled out. Due to the loss of oximetry data during the jump task, the exact degree of hypoxemia during the physical task remains unquantified. While the sample size was small, it comprised a large team of elite youth athletes training and competing together, minimizing inter-subject variability from sport or load differences. Regarding elite youth mogul ski athletes, our sample captures most of the target population, ensuring representativeness. However, results might not apply to different disciplines, to older athletes, to highlanders or acclimatized populations. Future studies should evaluate the dose-response effect of task duration at different altitudes, as duration might affect task sensitivity to fatigability. In the laboratory, there is also a need to evaluate the effect of cold combined with hypoxia, which is another environmental factor that could influence cognitive fatigability. The concurrent effects of physical fatigue and hypoxia cannot be fully disentangled in the present design; while this reflects ecologically valid conditions for athletes, future studies are required to clarify the causal inference regarding the independent role of hypoxia on cognitive performance. In the field, methodological questions include changes in response time with or without gloves and goggles, which might increase athlete comfort but might affect test results.

## Conclusions

In elite youth athletes, cognitive performance decreases in normobaric hypoxia (3500 m, SpO₂ ~ 88%) with potential effects on fatigability. This study also demonstrates that the 3-minute PVT and 10-minute cMSIT are promising tools for detecting environmental effects on cognitive fatigability in athletes, and it outlines methodological points critical for research in this area. These findings open the possibility of integrating cognitive tasks into athlete monitoring routines, allowing coaches to better detect overload or altitude-related impairments that might increase the risk of technical errors and injury.

## Supporting information

S1 File Properties of the Color Multisource Interference Task. (DOCX)

S2 File Jump task data by sex. (DOCX)

## Abbreviations

PVT: Psychomotor Vigilance Task
cMSIT: Colour Multisource Interference Task
PFC: PreFrontal Cortex
dACC: Dorsal Anterior Cingulate Cortex

## References

[1] Imray C, Booth A, Wright A, Bradwell A. Acute altitude illnesses. BMJ. 2011;343:d4943. doi: 10.1136/bmj.d4943 21844157

[2] Mitterwallner V, Steinbauer M, Mathes G, Walentowitz A. Global reduction of snow cover in ski areas under climate change. PLoS One. 2024;19(3):e0299735. doi: 10.1371/journal.pone.0299735 38478484 PMC10936838

[3] McMorris T, Hale BJ, Barwood M, Costello J, Corbett J. Effect of acute hypoxia on cognition: A systematic review and meta-regression analysis. Neurosci Biobehav Rev. 2017;74(Pt A):225–32. doi: 10.1016/j.neubiorev.2017.01.019 28111267

[4] Jiang Y, Cai K-E, Zhu L-L, Fan M, Zhao Y-Q, Wang D-M. A meta-analysis of multidimensional cognitive functions changes in different intensities of high-altitude hypoxia. Brain Behav. 2025;15(9):e70883. doi: 10.1002/brb3.70883 40976994 PMC12451029

[5] Jung M, Zou L, Yu JJ, Ryu S, Kong Z, Yang L, et al. Does exercise have a protective effect on cognitive function under hypoxia? A systematic review with meta-analysis. J Sport Health Sci. 2020;9(6):562–77. doi: 10.1016/j.jshs.2020.04.004 32325144 PMC7749263

[6] Skau S, Sundberg K, Kuhn H-G. A proposal for a unifying set of definitions of fatigue. Front Psychol. 2021;12:739764. doi: 10.3389/fpsyg.2021.739764 34721213 PMC8548736

[7] Enoka RM, Duchateau J. Translating fatigue to human performance. Med Sci Sports Exerc. 2016;48(11):2228–38. doi: 10.1249/MSS.0000000000000929 27015386 PMC5035715

[8] Hunter SK. Performance fatigability: Mechanisms and task specificity. Cold Spring Harbor Perspectives in Medicine. 2018;8:a029728. doi: 10.1101/cshperspect.a029728 PMC6027928 28507192

[9] Van Cutsem J, Marcora S, De Pauw K, Bailey S, Meeusen R, Roelands B. The effects of mental fatigue on physical performance: A systematic review. Sports Med. 2017;47(8):1569–88. doi: 10.1007/s40279-016-0672-0 28044281

[10] Diamond A. Executive functions. Annu Rev Psychol. 2013;64:135–68. doi: 10.1146/annurev-psych-113011-143750 23020641 PMC4084861

[11] Ochi G, Yamada Y, Hyodo K, Suwabe K, Fukuie T, Byun K, et al. Neural basis for reduced executive performance with hypoxic exercise. Neuroimage. 2018;171:75–83. doi: 10.1016/j.neuroimage.2017.12.091 29305162

[12] Varesco G, Staiano W, Bracco M, Pageaux B, Soulas L, Goisbault M, et al. Effects of 5-week brain endurance training on fatigue and performance in elite youth epée fencers. Int J Sports Physiol Perform. 2025;20(7):979–85. doi: 10.1123/ijspp.2024-0396 40404142

[13] De Wachter J, Roose M, Proost M, Habay J, Verstraelen M, De Bock S, et al. Prefrontal cortex oxygenation during a mentally fatiguing task in normoxia and hypoxia. Exp Brain Res. 2024;242(7):1807–19. doi: 10.1007/s00221-024-06867-y 38839618 PMC11208267

[14] Basner M, Mollicone D, Dinges DF. Validity and sensitivity of a brief psychomotor vigilance Test (PVT-B) to total and partial sleep deprivation. Acta Astronaut. 2011;69(11–12):949–59. doi: 10.1016/j.actaastro.2011.07.015 22025811 PMC3197786

[15] Basner M, Dinges DF. Maximizing sensitivity of the psychomotor vigilance test (PVT) to sleep loss. Sleep. 2011;34(5):581–91. doi: 10.1093/sleep/34.5.581 21532951 PMC3079937

[16] Basner M, Hermosillo E, Nasrini J, McGuire S, Saxena S, Moore TM. Repeated administration effects on psychomotor vigilance test performance. SLEEP. 2017;41. doi: 10.1093/sleep/zsx187 29126328

[17] O’Keeffe K, Raccuglia G, Hodder S, Lloyd A. Mental fatigue independent of boredom and sleepiness does not impact self-paced physical or cognitive performance in normoxia or hypoxia. J Sports Sci. 2021;39(15):1687–99. doi: 10.1080/02640414.2021.1896104 33678152

[18] Russell S, Jenkins D, Smith M, Halson S, Kelly V. The application of mental fatigue research to elite team sport performance: New perspectives. J Sci Med Sport. 2019;22(6):723–8. doi: 10.1016/j.jsams.2018.12.008 30606625

[19] Weiler H, Ennigkeit F, Spielmann J, Englert C. Increasing ecological validity in mental fatigue research-A Footbonaut study. Front Psychol. 2025;16:1586944. doi: 10.3389/fpsyg.2025.1586944 40497106 PMC12149105

[20] Bian C, Russell S, Mali A, Lathouwers E, De Pauw K, Habay J, et al. Methodological considerations and effectiveness for ecologically valid mental fatigue inducement in sports: A systematic review. Sports Med Open. 2025;11(1):82. doi: 10.1186/s40798-025-00891-0 40593374 PMC12214236

[21] Harwood B, Wright J, Burnet S. Reliability and validity of the Hypoxico Everest Summit II altitude generator. Proc Inst Mech Eng, Part P: J Sports Eng Technol. 2021;236:172–8. doi: 10.1177/1754337121995976

[22] Ochi G, Kuwamizu R, Suwabe K, Fukuie T, Hyodo K, Soya H. Cognitive fatigue due to exercise under normobaric hypoxia is related to hypoxemia during exercise. Sci Rep. 2022;12(1):9835. doi: 10.1038/s41598-022-14146-5 35764684 PMC9240057

[23] Aben B, Calderon CB, Bussche EV, Verguts T. Cognitive effort modulates connectivity between dorsal anterior cingulate cortex and task-relevant cortical areas. J Neurosci. 2020;40:3838–48. doi: 10.1523/jneurosci.2948-19.2020 32273486 PMC7204076

[24] Bush G, Shin LM, Holmes J, Rosen BR, Vogt BA. The multi-source interference task: Validation study with fMRI in individual subjects. Mol Psychiatry. 2003;8(1):60–70. doi: 10.1038/sj.mp.4001217 12556909

[25] Mohamed Zied K, Phillipe A, Pinon K, Havet-Thomassin V, Aubin G, Roy A, et al. Bilingualism and adult differences in inhibitory mechanisms: evidence from a bilingual stroop task. Brain Cogn. 2004;54(3):254–6. doi: 10.1016/j.bandc.2004.02.036 15050787

[26] Varesco G, Lavoie F-A, Adoutoro J, Michaud X, Guloy S, Martin A, et al. Acute effects of sleep extension on fatigue, inhibitory control, short-term vigilance and neuromuscular function in youth elite ice hockey players: A randomised crossover trial. J Sleep Res. 2025;:e70269. doi: 10.1111/jsr.70269 41431161 PMC13357769

[27] Dinges DF, Powell JW. Microcomputer analyses of performance on a portable, simple visual RT task during sustained operations. Behavior Research Methods, Instruments, & Computers. 1985;17(6):652–5. doi: 10.3758/bf03200977

[28] Arsintescu L, Kato KH, Cravalho PF, Feick NH, Stone LS, Flynn-Evans EE. Validation of a touchscreen psychomotor vigilance task. Accid Anal Prev. 2019;126:173–6. doi: 10.1016/j.aap.2017.11.041 29198969

[29] Grant DA, Honn KA, Layton ME, Riedy SM, Van Dongen HPA. 3-minute smartphone-based and tablet-based psychomotor vigilance tests for the assessment of reduced alertness due to sleep deprivation. Behav Res Methods. 2017;49(3):1020–9. doi: 10.3758/s13428-016-0763-8 27325169

[30] Hart SG. Nasa-Task Load Index (NASA-TLX); 20 Years Later. Proceedings of the Human Factors and Ergonomics Society Annual Meeting. 2006;50(9):904–8. doi: 10.1177/154193120605000909

[31] Haag EL, Weyand PG. Sex performance differences in vertical and horizontal jumping. R Soc Open Sci. 2025;12(9):241920. doi: 10.1098/rsos.241920 40994581 PMC12457033

[32] Wylie GR, Yao B, Genova HM, Chen MH, DeLuca J. Using functional connectivity changes associated with cognitive fatigue to delineate a fatigue network. Sci Rep. 2020;10(1):21927. doi: 10.1038/s41598-020-78768-3 33318529 PMC7736266

[33] Clairis N, Lopez-Persem A. Debates on the dorsomedial prefrontal/dorsal anterior cingulate cortex: Insights for future research. Brain. 2023;146(12):4826–44. doi: 10.1093/brain/awad263 37530487 PMC10690029

[34] Lambourne K, Tomporowski P. The effect of exercise-induced arousal on cognitive task performance: A meta-regression analysis. Brain Res. 2010;1341:12–24. doi: 10.1016/j.brainres.2010.03.091 20381468

[35] Venhorst A, Micklewright DP, Noakes TD. The psychophysiological determinants of pacing behaviour and performance during prolonged endurance exercise: A performance level and competition outcome comparison. Sports Med. 2018;48(10):2387–400. doi: 10.1007/s40279-018-0893-5 29532418

[36] Schmit C, Brisswalter J. Executive functioning during prolonged exercise: A fatigue-based neurocognitive perspective. International Review of Sport and Exercise Psychology. 2018;13(1):21–39. doi: 10.1080/1750984x.2018.1483527

[37] Sands WA, McNeal JR, Ochi MT, Urbanek TL, Jemni M, Stone MH. Comparison of the Wingate and Bosco anaerobic tests. J Strength Cond Res. 2004;18(4):810–5. doi: 10.1519/13923.1 15574087

[38] Takei N, Kakinoki K, Girard O, Hatta H. No influence of acute moderate normobaric hypoxia on performance and blood lactate concentration responses to repeated wingates. Int J Sports Physiol Perform. 2021;16(1):154–7. doi: 10.1123/ijspp.2019-0933 33120358

[39] Girard O, Brocherie F, Millet GP. Effects of altitude/hypoxia on single- and multiple-sprint performance: A comprehensive review. Sports Med. 2017;47:1931–49. doi: 10.1007/s40279-017-0733-z 28451905

[40] Martin K, Staiano W, Menaspà P, Hennessey T, Marcora S, Keegan R, et al. Superior inhibitory control and resistance to mental fatigue in professional road cyclists. PLoS One. 2016;11(7):e0159907. doi: 10.1371/journal.pone.0159907 27441380 PMC4956323

[41] Liu Y, Angstadt M, Taylor SF, Fitzgerald KD. The typical development of posterior medial frontal cortex function and connectivity during task control demands in youth 8-19years old. Neuroimage. 2016;137:97–106. doi: 10.1016/j.neuroimage.2016.05.019 27173761 PMC4916459

[42] Netzer NC, Rausch L, Eliasson AH, Gatterer H, Friess M, Burtscher M, et al. SpO2 and heart rate during a real hike at altitude are significantly different than at its simulation in normobaric hypoxia. Front Physiol. 2017;8:81. doi: 10.3389/fphys.2017.00081 28243206 PMC5303738

