# Supplementary material for: Assessment of cognitive performance and fatigability in elite athletes: Short and portable protocols for field monitoring under hypoxia
Source: PLoS One. 2026 Jul 10;21(7):e0353673. doi: 10.1371/journal.pone.0353673 (PMC13353968; doi:10.1371/journal.pone.0353673)
Supplement: S1 File — (DOCX) [file pone.0353673.s001.docx]

## S1. Properties of the Color Multisource Interference Task

The number of correct answers was highly correlated with the total trial accrued (r=0.98; p<0.001; Figure S1A). The relationship between the number of trials per minute and mean response time per minute was curvilinear (Equation 1; η^2^p=1.00; p<0.001; Figure S1B), i.e. changes in response time have nonlinear effects on total trials. In other words, when response time is slow, the total count is reduced disproportionately. There was no effect of time spent on task on this relationship (time effect: η^2^p=0.006; p=0.18).

*
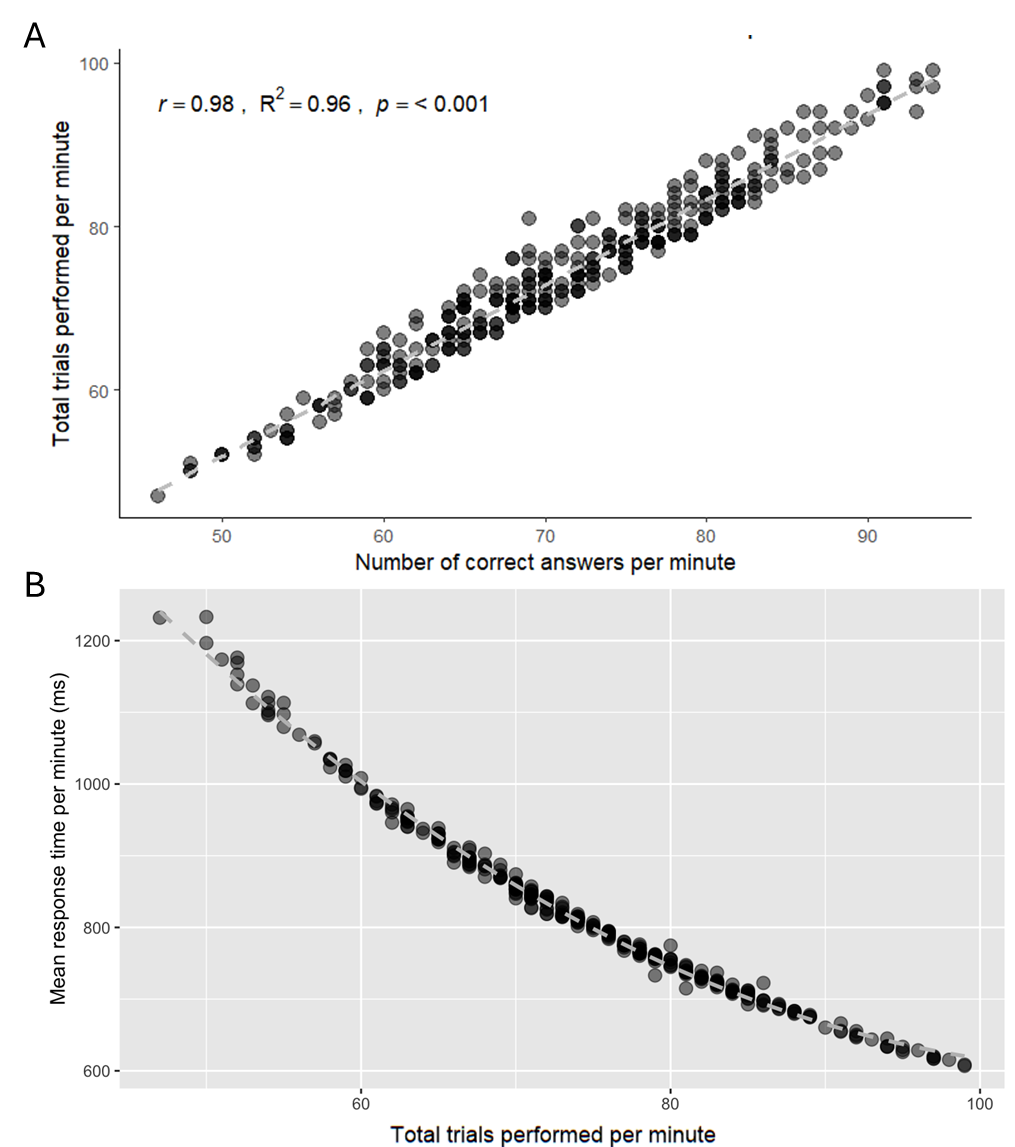
*

***Figure S1.*** *Panel A: Linear relationship between total number of trials performed and number of correct answers per minute during the 10 minutes colour multisource interference task. Panel B: Relationship between mean response time per minute and number of trial per minute.*

$$\hat{mean\_ResponseTime\_msᵢⱼ} = 826.372 - 2194.126 \times trials + 421.149 \times\mathrm{trials}^{2} + u₀ᵢ + \varepsilonᵢⱼ$$

*Equation S1.*

Similarly, the relationship between the number of correct answers per minute and mean response time per minute yielded a similar curvilinear relationship (η^2^p=0.91; p<0.001), with no time effect (η^2^p=0.01; p=0.83).

Several combinations showed positive or negative effects on response time (η^2^p=0.05; p<0.001). Faster responses are noted on combinations like 121 (-78.63 ms), 772 (-80.59 ms), 774 (-75.44 ms). Interestingly, several combination with specific pattern “XYX” showed to induce slower responses, and include combinations such as 232 (+83.45 ms), 242 (+75.11 ms), 252 (+73.09 ms), 262 (+109.43 ms), 282 (+85.52 ms), 292 (+72.94 ms), 363 (+145.75 ms), 383 (+153.90 ms), 393 (+133.09 ms), 565 (+191.51 ms), 575 (+110.09 ms), 585 (+129.35 ms), 595 (+104.83 ms), and several others with a similar pattern (all p<0.05; Figure S2). No combination×condition interaction was found (η^2^p=0.009; p=0.54), showing appropriate randomization across conditions.

Compared to pattern “XXY” or “YXX”, the pattern “XYX” probably involve repetitive saccades to compare numbers separated by another one (e.g. [1]).


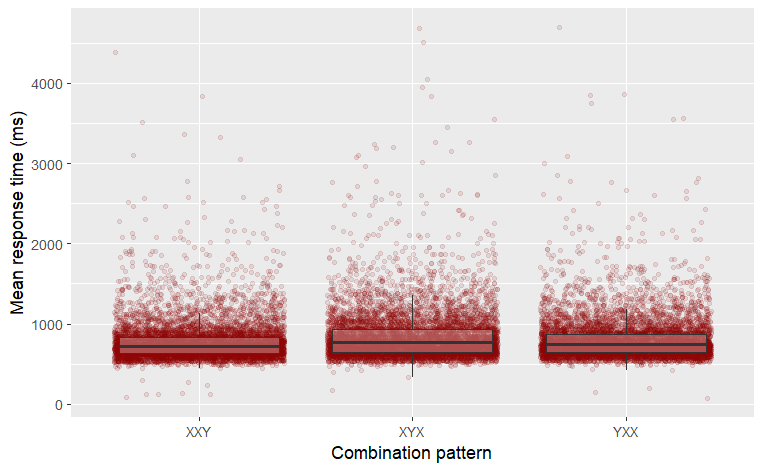


***Figure S2.*** *Boxplot and individual trial data divided across pattern.*

Reference

1. Pearson DG, Ball K, Smith DT. Oculomotor preparation as a rehearsal mechanism in spatial working memory. Cognition. 2014;132: 416–428. doi:10.1016/j.cognition.2014.05.006
