# Supplementary material for: Assessment of cognitive performance and fatigability in elite athletes: Short and portable protocols for field monitoring under hypoxia
Source: PLoS One. 2026 Jul 10;21(7):e0353673. doi: 10.1371/journal.pone.0353673 (PMC13353968; doi:10.1371/journal.pone.0353673)
Supplement: S2 File — (DOCX) [file pone.0353673.s002.docx]

## S2. Jump task data by sex

**Table S1.** Jump data across conditions, sets and sexes.

|  | |  | | ***Normoxia*** | | | ***Hypoxia*** | | |
| --- | --- | --- | --- | --- | --- | --- | --- | --- | --- |
| ***Men (n=10****)* | | | | ***Number*** | ***Height (cm)*** | ***Impulse (N s)*** | ***Number*** | ***Height (cm)*** | ***Impulse (N s)*** |
| *set* | | *1* | 52±4 | 21.2±5.7 | 21674±2600 | 52±7 | 20.8±6.4 | 20804±2772 |  |
|  |  | *2* | 48±4 | 22.2±4.1 | 20470±2397 | 51±7 | 21.7±6.8 | 20807±2609 |  |
|  |  | *3* | 49±5 | 23.3±4 | 21468±3268 | 50±8 | 22±6.4 | 20467±2591 |  |
| ***Women (n=7)*** | | | |  |  |  |  |  |  |
| *set* | | *1* | 55±4 | 16.2±3.5 | 17340±1672 | 52±4 | 17.8±3.3 | 18015±1533 |  |
|  |  | *2* | 54±3 | 16.4±4.2 | 17464±1855 | 53±3 | 16.9±3.3 | 17840±1453 |  |
|  |  | *3* | 56±5 | 16.5±4.2 | 18345±1417 | 52±3 | 16.7±2.6 | 18486±1762 |  |

For impulse, sex did not interact with condition×set (sex×condition×set: η^2^p=0.07; p=0.13), nor with condition (condition×sex: η^2^p<0.001; p=0.99), or set (sex×set: η^2^p=0.09; p=0.66), but overall women showed lower total impulse than men (sex main effect: η^2^p=0.36; p=0.015), which was expected.

Previous studies have shown that body mass, rather than binary sex, provides superior prediction of jump performance outcomes [1] . Thus, we compared the variance explained by models including sex versus body mass. A linear mixed-effects model was fitted to examine the effects of condition, series, and covariates on jump impulse, with participant ID included as a random intercept. The model including sex as a fixed covariate [impulse ~ condition + set + sex + condition:set + (1 | ID)] resulted in an AIC of 1380.7 and deviance of 1362.7. Replacing sex with body mass significantly improved model fit [impulse ~ condition + set + body mass + condition:set + (1 | ID); AIC = 1359.6, deviance = 1341.6], as evidenced by a likelihood ratio test (χ² = 21.06, p < 0.001). This indicates that body mass explains substantially more variability in jump impulse than sex, enhancing model precision for jump analysis.

We also expressed jump height and impulse normalized by body mass (Table S2). Similarly to not normalized data, sex did not interact with condition×set (sex×condition×set: η^2^p=0.07; p=0.15), nor with condition (condition×sex: η^2^p<0.001; p=0.63), or set (sex×set: η^2^p=0.10; p=0.051), and the main effect of sex disappeared (η^2^p=0.02; p=0.64).

**Table S2**. Jump data across conditions, sets and sexes normalized by body mass.

|  | | | ***Normoxia*** | | ***Hypoxia*** | |
| --- | --- | --- | --- | --- | --- | --- |
| ***Men (n=10****)* | | | ***Height (cm / kg)*** | ***Impulse (N s / kg)*** | ***Height (cm / kg)*** | ***Impulse (N s / kg)*** |
| *set* | *1* | 0.292±0.096 | 293±9 | 0.291±0.1 | 287±10 |  |
|  | *2* | 0.306±0.078 | 277±15 | 0.304±0.11 | 287±7 |  |
|  | *3* | 0.321±0.08 | 290±22 | 0.308±0.1 | 282±9 |  |
| ***Women (n=7)*** | | |  |  |  |  |
| *set* | *1* | 0.266±0.055 | 284±17 | 0.282±0.059 | 280±25 |  |
|  | *2* | 0.269±0.063 | 285±11 | 0.267±0.06 | 277±20 |  |
|  | *3* | 0.272±0.066 | 301±17 | 0.256±0.051 | 279±13 |  |

References

1. Haag EL, Weyand PG. Sex performance differences in vertical and horizontal jumping. R Soc Open Sci. 2025;12: 241920. doi:10.1098/rsos.241920
