# Supplementary material for: Assessment of cognitive performance and fatigability in elite athletes: Short and portable protocols for field monitoring under hypoxia
Source: PLoS One. 2026 Jul 10;21(7):e0353673. doi: 10.1371/journal.pone.0353673 (PMC13353968; doi:10.1371/journal.pone.0353673)
Supplement: Attachment — Submitted filename: Response to reviewers_R2.docx [file pone.0353673.s005.docx]

Dear editor,

Thank you for letting us revise our manuscript and providing additional time to do so.

Response to all comments of the reviewers are provided in the current letter and all changes have been marked in red in the revised manuscript.

To address the comments regarding our design’s limitations, we ran a second data collection (called in the manuscript “Study II”), where we addressed the main limitations of the study, i.e. the non-randomized sessions and the inability to separate the effect of jump task effect on cognitive performance. To address these limitations and improve internal validity (i.e. running our own replication study), we collected data one year later on the same team during the pre-season period. Athletes were tested 3 weeks apart and assigned to two groups balanced for age and sex but otherwise randomized. Procedures remained similar to the previous data collection (familiarization, baseline testing, and cognitive testing), but one group performed cognitive testing in hypoxia during the first session while the other did so during the second session, in a crossover design. No jump task was performed, isolating hypoxia’s effect on cognitive performance. Athletes were tested in the same order and time across sections. The manuscript has been modified accordingly and now better support the initial findings, evidencing how the cognitive task was able to detect hypoxia-related impairments in cognitive function. We hope that we addressed all the concerns of the reviewers from the previous and current round. The revised abstract summarizes the results.

*“Purpose: This study aimed to evaluate whether short (~15 min) cognitive protocols can detect fatigability in normoxia versus normobaric hypoxia in elite youth athletes, using tasks directly transferable to the field.*

*Methods: Elite youth mogul skiers (18±2 y) completed two studies. In Study I, 17 athletes (7 women) performed a cognitive test after a repeated tuck-jump protocol designed to mimic mid-training physical demands under two conditions in fixed order: sham normoxia (simulated 200 m) and normobaric hypoxia (3500 m, blood saturation ~88%). In Study II, 16 athletes (6 women) performed the same cognitive test without the jump task, with conditions randomized. In both studies, response time and accuracy during a 10-min color Multisource Interference Task (cMSIT) were used to assess cognitive performance, and a 3-min Psychomotor Vigilance Task (PVT) was administered before and after the cMSIT to quantify fatigability. Cognitive tests were performed on portable tablet devices.*

*Results: After adjusting for baseline cognitive performance, cMSIT response time was greater in hypoxia than in normoxia in both Study I (10±2%, p=0.004) and Study II (4±1%, p<0.001). Accuracy remained high and similar across conditions in both studies (Study I: 96±3%; Study II: 96±0.5%; p>0.09). In Study I, PVT response time increased only in hypoxia, from 363±74 ms to 375±89 ms (p=0.021), and lapses increased from 1±0.5 to 3±2.5 (p<0.001), whereas lapses remained unchanged in normoxia and in Study II (p>0.82). In Study I, jump task performance showed a condition×set interaction (p=0.048), with lower performance in the third set in hypoxia (19806±2464 N·s) than in normoxia (20130±3016 N·s; p=0.012).*

*Conclusions: In elite youth athletes, hypoxemia reduces cognitive performance and could lead to increased fatigability. Importantly, hypoxia-related impairments are detectable with short portable cognitive assessments, which are promising tools for field monitoring, particularly when tasks combines cognitive and physical demands.”*

Reviewer #1: General Comments

Major Weaknesses:

Physiological Validation: The failure to collect usable heart rate and blood oxygen saturation data during the jump task due to sensor displacement limits the physiological validation of the hypoxic stimulus during the actual physical effort. Relying solely on resting data is a limitation that must be addressed more prominently.
Statistical Covariates: The rationale for replacing sex with body mass in the linear mixed-effects models requires stronger physiological justification within the main text. Sex differences in neuromuscular and cognitive fatigability are well-documented and extend beyond simple anthropometric differences.

Thank you for your comments, we addressed these points in the response to the specific comments, and we added more information on the limitation section and the statistical analysis section to address the reviewer major concerns.

Please note that, considering this round and the previous round of comments and the major limitation our design had in investigating our hypothesis, we ran a second data collection (called in the manuscript “Study II”), where we addressed the main limitations of the study, i.e. the non-randomized sessions and the inability to separate the effect of jump task effect on cognitive performance. To address these limitations and improve internal validity, we collected data one year later on the same team during the pre-season period. Athletes were tested 3 weeks apart and assigned to two groups balanced for age and sex but otherwise randomized. Procedures remained similar to the previous data collection (familiarization, baseline testing, and cognitive testing), but one group performed cognitive testing in hypoxia during the first session while the other did so during the second session, in a crossover design. No jump task was performed, isolating hypoxia’s effect on cognitive performance. Athletes were tested in the same order and time across sections. The manuscript has been modified accordingly and now better support the initial findings, evidencing how the cognitive task was able to detect hypoxia-related impairments in cognitive function. Thank you again for your comments that allowed us to improve our manuscript, we hope that we addressed all the concerns of the reviewer from the previous and current round.

Minor Weaknesses:

Terminology: The manuscript occasionally interchanges "hypoxia" (environmental condition) and "hypoxemia" (physiological state). Strict differentiation is required.

Thank you for your comment. We were very careful in using hypoxia when referring to the environmental condition imposed, and hypoxaemia when considering the physiological state driving consequences in physical and cognitive performance. We double-checked the use of hypoxia and hypoxaemia across the manuscript.

Formatting and Typography: There are several typographical errors, missing punctuations in statistical reporting, and inconsistent formatting in Table 1 that require correction.

Thank you, we completely proofread the manuscript.

Specific Comments

Page 2, Line 64: Specify the number of male athletes. You list "$n=17$, 7 women," but the male count should be explicit for immediate clarity.

We respectfully decided to keep the information as presented, if over 17 participants 7 are biological women, we prefer to avoid redundancy specifying that the remaining 10 are biological males.

Page 2, Line 82: Insert "a" before "condition set interaction" and use the standard notation "condition $\times$ set".

We apologize if we are missing a detail, but we were unable to spot a missing “×” across the manuscript.

Page 4, Line 124: Change "refers as" to "is defined as" or "refers to".

Thank you, this has been corrected.

Page 6, Line 227: There is a typographical error and a missing closing parenthesis in the Brugmann scale reporting: "physical item 3 2)". Correct the value and add the parenthesis.

We apologize if we are missing a detail but the manuscript reads:
“excessive daytime fatigue (Brugmann scale: mental item=3±1, physical item=3±2),”

Page 8, Line 282: The use of a sham condition (200 m) involves participant deception. Specify in the ethical procedures section if a debriefing occurred post-study.

Thank you for this important comment. Yes, a debrief occurred and feedback was provided. The manuscript now reads: *“Due to the use of a sham condition (200 m), participants were not informed of the true altitude exposure until after data collection. All participants were debriefed post-study, and feedback regarding the study procedures and aims was provided.”*

Page 11, Line 396: The loss of oximetry data during the jump task is briefly mentioned. Add a sentence in the Limitations section acknowledging that the exact degree of hypoxemia during the physical task remains unquantified.
Thank you, we added to the limitations: Due to loss of oximetry data during the jump task, the exact degree of hypoxemia during the physical task remains unquantified.

Page 11, Line 427: Adjusting for body mass instead of sex is a specific analytical choice. Add a brief sentence in this section justifying why body mass is a superior predictor for this specific youth mogul skier cohort, rather than referring solely to the supplement.
Thank you, we modified the sentence to better reflect our rationale and analyses:
Previous studies have shown that body mass (continuous variable), rather than binary sex, provides superior prediction of jump performance outcomes [30] (see Supporting Information S2 for demonstration and data divided by sex). For cognitive testing, the sex factor was removed from the models as it did not affect the results (all p>0.07).

Page 12, Line 447: The phrase "influenza, different individuals" is grammatically awkward. Revise to "due to influenza (different individuals)."

Thank you, we modified the manuscript as suggested.

Page 13, Line 475: Missing a semicolon or comma before the p-value in the accuracy data: "$(97\pm2\% \eta^{2}p=0.11 p=0.18)$".
We apologize if we are missing a detail but we were unable to spot a missing semicolon or comma. The manuscript reads: “No difference in cMSIT accuracy was found between conditions at baseline (97±2%; η^2^p=0.11; p=0.18).”

Page 13, Line 489: Missing the subscript "$p$" for partial eta squared: "$\eta^{2}<0.001$ $p=0.98$".
Thank you, we modified the manuscript as suggested.

Page 14, Line 521 (Table 1): The formatting is inconsistent. The response time cells for Hypoxia and Normoxia lack the standard deviation symbol (e.g., "363 74" should be "363 $\pm$ 74").

We apologize if we are missing a detail, but we were unable to spot a missing “±”. Table 1 reads “363±74”

Page 16, Line 604: Change "different effort put in the task" to "differences in effort exerted during the task".
Thank you, we modified the manuscript as suggested.

Page 18, Line 676: Specify the exact statistical threshold or method used to determine the curvilinear association mentioned.
Thank you, added the term “quadratic” to detail the type of curvilinear association that we expected.

Page 19, Line 712: Change the comma to a period or semicolon before "while this reflects ecologically valid conditions" to correct the run-on sentence.

Thank you, we modified the manuscript as suggested.

Reviewer #2: I would like to thank the authors for their thorough and thoughtful revision of the manuscript. The responses provided demonstrate a clear effort to address the reviewers’ comments, and several aspects of the manuscript have been improved, particularly in terms of clarity, structure, and justification of methodological choices.
That said, I would like to highlight a few remaining concerns that, in my view, would benefit from further clarification and strengthening.
Thank you for your comments that helped us improving our manuscript. All comments have been addressed and modifications/addition marked in red.

First, regarding the blinding procedure, although the authors provide a rationale for not including a manipulation check, the absence of any assessment of participants’ perception of the experimental condition remains a relevant limitation. Given the nature of hypoxia and the population studied, this issue should be more explicitly acknowledged in the manuscript as a potential source of bias affecting internal validity.
To address the reviewers comments regarding our design’s limitations, we ran a second data collection (called in the manuscript “Study II”), where we addressed the main limitations of the study, i.e. the non-randomized sessions and the inability to separate the effect of jump task effect on cognitive performance. To address these limitations and improve internal validity (i.e. running our own replication study), we collected data one year later on the same team during the pre-season period. Athletes were tested 3 weeks apart and assigned to two groups balanced for age and sex but otherwise randomized. Procedures remained similar to the previous data collection (familiarization, baseline testing, and cognitive testing), but one group performed cognitive testing in hypoxia during the first session while the other did so during the second session, in a crossover design. No jump task was performed, isolating hypoxia’s effect on cognitive performance. Athletes were tested in the same order and time across sections. The manuscript has been modified accordingly and now better support the initial findings, evidencing how the cognitive task was able to detect hypoxia-related impairments in cognitive function.

Second, the operationalization of cognitive fatigability, particularly the use of pre–post changes in PVT performance following the cMSIT, is better justified in the revised version. However, the conceptual distinction between the inducing task and the outcome measure could still be more clearly articulated, especially considering the broader theoretical debate around fatigability assessment.

Thank you for evidencing this important aspect. Fatigability in the present work (and in accordance with a large part of the literature) represents the decline in a given performance outcome over a specific amount of time in the case of constant-load tasks. When the load is not constant, or the effort provided is not constant (depending on the task), it becomes arguably inaccurate to assess fatigability solely on the base of performance changes over time. Thus, a common strategy is the use of a “confirmation” test performed before and after the task. The cMSIT is designed to stress inhibitory control and offers the advantage to let the athlete to “pace”, that is, change the amount of effort put into the task completion based on cognitive resources available to achieve the goal, i.e. accruing as many correct trials as possible. Combining the results of Study I and Study II, it appears that i) the presence of a physical task before the cognitive task exacerbate the effect of hypoxia in reducing cognitive performance and increasing fatigability; ii) in the presence of the physical task, individuals might be pre-fatigue, and might adopt a more conservative pacing strategy, so performance across the cMSIT did not decline over time but fatigability was observed via changes pre- to post-cMSIT in PVT performance; and iii) the cognitive task alone, without pre-fatigue, let athletes to optimize pacing strategy to achieve a better performance over time but the effect on fatigability are arguably reduced.

Third, the duration of the cMSIT (10 minutes) remains a critical point. The absence of a time effect within the task raises important questions about its effectiveness as an inducer of cognitive fatigability. While this is acknowledged, I believe this limitation should be more strongly emphasized and more directly discussed in relation to the validity of the fatigability construct used in the study.

Thank you for evidencing this important point. We now better address this issue in the discussion:

*“These results also suggest that time on task was too short to induce large impairments in our experimental conditions, despite the high cognitive resources mobilized in both normoxia and hypoxia, as shown by the results of the NASA-TLX questionnaire. This is not surprising as athletes present low fatigability compared to non-athletes [40], and longer task durations might be needed. For instance, in a previous study we observed that a 30-minute cMSIT was sufficient to consistently induce impairments in their PVT performance [26]. Our results underline the need to test for different outcomes that might impact fatigability and performance in real-world scenarios, such as inhibitory control and sustained attention, which both have meaningful implications for athletes in decision-making, technical execution, and safety.*

*Considering our results, ignoring the effect of hypoxia when planning training at altitude could result in increased cognitive fatigability, which might influence negatively the risk of technical errors and thus injuries, especially across multiple days of training.”*

And further addressed by the study 2 part:

*“Interestingly, with prior physical exercise we also observed i) no change in performance across the task, and ii) a pre- to post-PVT impairment in hypoxia only. In Study I, the presence of a condition effect in cMSIT performance might be interpreted as differences in effort put into the task between sessions, however, a time×condition interaction for cMSIT could be expected in this case. Study II confirmed these results in a different context: without prior exercise, a decline in cMSIT performance was observed with time, without difference across conditions nor changes in PVT performance from pre- to post-cMSIT.*

*Physical exercise prior to the cognitive task likely altered arousal and effort-regulation processes, thereby modulating performance (pacing) strategy for the cognitive task, while the absence of prior exercise may have reduced this regulation process or arousal, allowing greater time-dependent decline in cMSIT performance [34,35].”*

We further added in the limitation section that “Future studies should evaluate the dose-response effect of task duration at different altitudes, as duration might affect task sensitivity to fatigability”.

Finally, I would also encourage the authors to further reinforce the discussion around the interpretative complexity introduced by the inclusion of a physical task prior to cognitive assessment. Although this choice enhances ecological validity, it also limits the ability to disentangle the independent effects of hypoxia and physical fatigue, and this should be clearly and critically reflected in the discussion.
To address the reviewers comments regarding our design’s limitations, Study II did not include the jump task, to better isolate the effect of hypoxia on cognitive performance.

Addressing these points would, in my opinion, further strengthen the manuscript and improve its conceptual clarity and methodological transparency.
Thank you for your kind words and the insightful comments that helped us improving our manuscript.
