# Supplementary material for: Assessment of cognitive performance and fatigability in elite athletes: Short and portable protocols for field monitoring under hypoxia
Source: PLoS One. 2026 Jul 10;21(7):e0353673. doi: 10.1371/journal.pone.0353673 (PMC13353968; doi:10.1371/journal.pone.0353673)
Supplement: Attachment — Submitted filename: Reviewer_response_R1.docx [file pone.0353673.s004.docx]

Reviewer #1: General Comments

The manuscript investigates the application of a short cognitive and physical testing protocol to assess cognitive fatigability under normobaric hypoxia in elite youth athletes. While the objective aligns with current needs in sports science for field-deployable monitoring tools, the study design and execution present significant methodological challenges that compromise the integrity of the findings.

We would like to thank the reviewer for his kind words and his observations that allowed us to improve our manuscript. We addressed all reviewers’ comments, and all changes to the manuscript are marked in red.

Major Weaknesses:

Fixed-Order Crossover Design: The most critical flaw is the lack of randomization in the condition sequence. All participants completed the normoxia session first, followed by the hypoxia session two weeks later. In cognitive assessments involving inhibitory control and psychomotor vigilance, learning, practice, and habituation effects are substantial. Consequently, the effect of hypoxia is inherently confounded by the sequence effect. The baseline differences observed in cMSIT performance strongly indicate a practice effect from session 1 to session 2.

*Thank you for this relevant comment, we acknowledge this limitation in our design, which was driven by practical needs: the hypoxic generator needs approximately 2h to ensure reliable stabilization of altitude, making it impossible to switch from low to high altitude promptly and thus making it unfeasible to divide athletes in two groups. Testing athletes on different days was also not a viable choice due to logistical constraints and, importantly, by the fact that testing athletes at different days would induce training to act as confounder; it is thus essential to test athletes from the same team the same day. This has been acknowledged at Lines 172–178.*

*We want to underline that PVT has been selected to test for fatigability also because it has consistently shown to not have a familiarization/learning/practice effect along a wide range of literature studies (e.g. Basner 2011). We selected the cMSIT with zero-lag between trials over other inhibitory control tasks (e.g. Stroop task, Simon task, Flanker task) as the specific properties of the task (detailed in supplemental materials S1) make the task less subject to familiarization effect. Finally, whether differences in baseline between sessions were due to familiarization, cognitive performance or the combination of both, was addressed by controlling for baseline results in our analysis. Thus, if as noted by the reviewer, there was a “baseline differences observed in cMSIT performance strongly indicate a practice effect from session 1 to session 2”, this was accounted for in our lmm.*

*These limitations have been acknowledged at Lines 437–445: “The main limitation of the present study is that sessions were not randomized: timely switching between conditions (a prerogative of randomization) was not possible as the hypoxic chamber requires hours to stabilize at new altitudes. To reduce the order effect, we used a sham condition (200 m) and made the athletes naïve of the study true objective. Non randomization could influence baseline cMSIT performance. We were aware of the possible learning, fatigue, or expectation effects before the study and adopted all available procedures to adress this limitation, such as performing an extensive familiarisation in both sessions, using tasks with limited learning effect and contolling for baseline cognitive performance. However, the potential impact of this limitation on the internal validity of the findings cannot be completely ruled out.”*

Missing Data and Sample Size: The initial sample size is small ($n=17$), which is typical for elite populations, but subject attrition reduces the analytical power further. Missing physiological data (heart rate and SpO2 during the jump task) due to equipment issues represents a significant loss of objective internal load metrics required to validate the physiological strain of the task.

*Thank you for your comment. Indeed, it is difficult to recruit large samples of elite athletes, especially from winter sports, whose teams rarely surpass the 10 members. One main advantage is that recruiting elite individuals of the same team would largely reduce possible interindividual confounders, minimizing those related to lifestyle or baseline fitness, which are a drawback in several studies evaluating active individuals (e.g. Van Cutsem et al. 2017; Martin et al. 2016]). Optimising modelling for small sample sizes is not an easy feat, but we believe our lmm could address several objections related to insufficient statistical power (e.g., Lakens, 2022). Finally, attrition was low as the totality of the participants took part in the protocol, leaving out only a couple of individuals who could not take part to testing due to influenza or injury (detailed at Lines 271–272).*

*We do not think that lack of SpO2 and HR data during the physical task represent a major drawback as external load was captured in the hop test (a simil-time trial / sprinting task), physiological strain should result in performance decline, as we observed in hypoxia. Furthermore, this limitation might address secondary outcomes, while the hop test goal was to mimic a mid-training situation (Lines 117–120 and 403-411).*

App Validation: The study relies on a custom-developed iOS application for cMSIT and PVT. Peer-reviewed validation of this specific application against established gold standards (e.g., PC-based E-Prime or standard PVT-192 hardware) is absent from the manuscript.

*We discuss this point at Lines 203–221. In a nutshell, previous app attempting to validate task using a cross-device approach failed as the device architecture was systematically different. Some adjusted the difference manually (e.g. subtracting a fix ~77 ms from the result app to improve precision; Arsintescu et al. 2019). As such, we re-validated the original construct (lapses and reaction time; Basner et al. 2011, 2017) and discussed the implication of re-adjustment vs. construct evaluation at Lines 414-423. We are convinced that this is a better approach than adjusting the sensitivity of the device to another just by re-centering a median bias.*

Minor Weaknesses:

Blinding Efficacy: The authors state participants were naïve to the condition. However, symptoms of hypoxemia (SpO2 ~88%) during physical exertion would likely unblind elite athletes attuned to their physiological states. No manipulation check was reported to confirm if blinding was successful.

*Thank you for your comment. To ensure blinding, we opted to make athletes naïve of the whole experimental conditions. This decision resulted of course in the inability to make a manipulation check. That is, we told athletes that both conditions were at altitude, assuming a 2 weeks recall is most likely biased, so interoceptive comparison across sessions was likely believed by the athletes themselves to be inaccurate (e.g. Allocco et al. 2025; Birol et al. 2024). That is, athletes were aware that they could not reliably compare the two sessions, and this uncertainty played in our favor, maximizing blinding when telling them that both sessions were in hypoxia (described at Lines 130-131 and 171–178 and 439-440 in the limitations).*

Statistical Reporting of Count Data: Table 1 reports count data (lapses) with means and standard deviations, despite the authors explicitly acknowledging a right-skewed Poisson distribution.

*We respectfully would like to point out that, as explicitly stated in the table notes, Lapses were indeed presented as median±interquartile range, not as mean±SD, a defined by the annotation “^a^”. , we apologize it is was not sufficiently highlighted. We have further highlighted this in the table notes in the revised version.*

Specific Comments

Page 9, Lines 100-101: The title asserts that the protocols "detect hypoxia effects," which is a deterministic claim given the fixed-order design. Reframing the title to reflect the exploratory nature of the study would mitigate definitive claims regarding hypoxia detection.

*Thank you for your comment, the reviewer indicated lines 100-101, which mention “Using*

*short, portable tasks would allow for valid and repeated assessments of cognitive fatigability in training*

*conditions, addressing the growing need for appropriate daily monitoring in the field.” We believe the reviewer indicates the title should better reflect this statement avoiding this deterministic claim. Thus, we modified the manuscript title in “Objective assessment of cognitive fatigability in elite youth athletes: short portable protocols for field monitoring under hypoxia”*

Page 14, Lines 132-144: A formal a priori power analysis or sample size justification is absent. Providing an a priori statistical justification would determine if the study was adequately powered to detect condition $\times$ time interactions amid high inter-individual variability.

*We recognize that power analysis should be population-specific.Because no prior study has examined comparable outcomes in elite youth moguls or freestyle ski athletes, an a priori power calculation based on an external effect size would have been speculative. We therefore added a sample size justification based on the rarity of the population, the feasibility of recruiting this cohort, and the repeated-measures design, which improves statistical sensitivity within participants. We prefer not to perform post hoc power analysis to prove adequacy as observed power is largely a function of the p-value and the sample effect estimate (Lakens 2022). Our within-subject repeated-measures structure (multiple assessments per participant across conditions and time points) increased statistical sensitivity and power relative to between-subject designs, even with modest sample size typical of sport science, by reducing inter-individual variability and leveraging participant-specific baselines.* *This approach aligns with recommendations for exploratory studies in specialized populations where population-specific effect sizes are unavailable (Lakens 2022). We have further clarified that our sample size was determined by convenience sampling and it represented the ~5% of the total population, which is large (Lines 139-141). This is further discussed in the limitations at Lines 445–449.*

Page 14, Lines 147-148: A testing window spanning three and a half hours introduces potential circadian variation in cognitive fatigability. Controlling for exact time-of-day testing would isolate the hypoxic effect from circadian cognitive fluctuations [Amor SB, Dhahbi W, Bougrine H, Bessifi M, Geantă VA, Ursu VE, Trabelsi K, Souissi N: Differential Time-of-Day Effects of Caffeine Capsule and Mouth Rinse on Cognitive Performance in Adolescent Male Volleyball Athletes: A Randomized Crossover Investigation. Life 2025, 16(1):33].’

*Thank you for this relevant comment. Following the reviewer’s suggestion, we adjusted for hour of testing all our outcomes. The adjustment did not influence PVT (all p>0.19), cMSIT accuracy (p=0.95) or jump performance (p=0.10) and thus removed from the model as per the Occam razor principle of parsimony, avoiding overfitting (Burnham & Anderson, 2002). However, it had an effect on cMSIT response time (all p<0.02) and thus kept in the model. While this did not influence the results, it changed slightly the estimates, overall improving the precision of the model and confirming our result.*

*The statistical analysis section (Lines 257-259 “We adjusted for hour of testing our estimates. Since it had no effect on PVT, cMSIT accuracy or jump performance (all p≥0.10), this term was removed from the model (Occam razor principle). However, it was kept for cMSIT response time (all p<0.02).”) and the result section (Lines 296–300) have been modified accordingly. It changed also the estimated difference from hypoxia and normoxia. Now hypoxia was estimated to be ~10% worst than normoxia. This result has been adjusted in the abstract as well. We adjusted Figure 3 according to the new estimates. Also, the discussion now reads (L386-391): “The unadjusted ~7% faster response time in hypoxia would reflect pre-existing inter-session baseline differences rather than a true performance advantage. When controlling for baseline cMSIT performance, hypoxia was associated with ~10% slower performance, indicating that the covariate adjustment was essential to isolate the true condition effect from session-to-session variability, particularly in elite youth athletes whose cognitive performance may fluctuate with training and academic load.”*

Page 14, Lines 171-173: The protocol relies on a sham altitude (200m) to maintain blinding. Reporting post-session surveys regarding perceived physical strain would verify the actual efficacy of this blinding strategy.

*Unfortunately, we did not record perceived physical strain. We will keep this suggestion in mind for future studies, thank you.*

Page 15, Lines 175-179: The justification for the non-randomized design relies purely on logistical convenience (chamber stabilization time). Providing secondary statistical analyses comparing session 1 vs. session 2 learning trajectories in a control group would clarify the sequence effect.

*We do not fully understand what should be compared to what control group as there was no such group in our study, but these limitations have been acknowledged at Lines 437–445:”* *The main limitation of the present study is that sessions were not randomized: timely switching between conditions (a prerogative of randomization) was not possible as the hypoxic chamber requires hours to stabilize at new altitudes. To reduce the order effect, we used a sham condition (200 m) and made the athletes naïve of the study true objective. Non randomization could influence baseline cMSIT performance. We were aware of the possible learning, fatigue, or expectation effects before the study and adopted all available procedures to adress this limitation, such as performing an extensive familiarisation in both sessions, using tasks with limited learning effect and contolling for baseline cognitive performance. However, the potential impact of this limitation on the internal validity of the findings cannot be completely ruled out.”*
*We want to underline that if as noted by the reviewer, there was a “baseline differences observed in cMSIT performance strongly indicate a practice effect from session 1 to session 2”, this was accounted for in our lmm.*

Page 16, Lines 198-212: The custom iOS application lacks formal validation against standard hardware, and touchscreen latencies vary significantly by device. Detailing the internal latency of the iPad model would demonstrate that touchscreen polling rates do not artificially skew the PVT response times. Furthermore, establishing a framework for technological validity would clarify the reliability of the derived response times [Dhahbi W, Chamari K: The Algorithmic Athlete: A Call to Standardize Assessment of Sensor Technologies and Artificial Intelligence. International Journal of Sports Physiology and Performance 2026, 1(aop):1-2].

*We added iPad model specifications and latency details (lines 221-224): “The tablet used (iPad 10th generation, 2022, A14 Bionic; iOS 18.7.7) featured a 60 Hz touchscreen polling (~16.7 ms interval) and end-to-end latency of 70-90 ms per benchmarks, which justifies critical re-evaluation of threshold for lapses that we performed following the rationale presented in Basner et al. [16] original PVT work”. Furthermore, as abovementioned, we re-validated the original construct (lapses and reaction time) and discussed the implication of re-adjustment vs. construct evaluation (L414-434).. We proceeded as the original works evaluating lapses threshold for the PVT-192 device (Basner 2011).*


Page 16, Lines 223-228: Utilizing repeated tuck jumps exclusively as the physical fatiguing mechanism neglects the complex multi-joint strength profiling necessary to fully assess lower-body endurance under hypoxia. Acknowledging the multi-dimensional nature of vertical jump performance would provide a more precise biomechanical context for the observed decrement in the third series [Sever O, Ceylan Hİ, Dhahbi W, Yilmaz HH, Özdemir K, Yazici AG, Jalalov F, Günay AR, Bayrakdaroğlu S, Stefanica V: Multi-joint isokinetic strength profiling as a predictor of vertical jump performance in elite freestyle wrestlers: A cross-sectional principal component analysis. Medicine 2026, 105(2):e47084].

*We thank the reviewer for highlighting the multi-joint complexity of vertical jump performance. However, repeated tuck jumps were specifically selected as an ecologically valid fatiguing protocol for moguls skiers, mimicking the high-repetition aerial landings and rapid Stretch-Shortening Cycle demands of mogul courses (>2 hit/sec over 30s runs), and deemed an appropriate protocol efficacy for our field-based hypoxia study (e.g. Weiler et al. 2025; Bian et al. 2025). While comprehensive isokinetic profiling would provide deeper biomechanical insight, we believe our focus on mid-training simulated fatigue induction (not strength profiling) justifies this targeted approach. At the same time, it might not by design be possible to separate the effet of the jump task from the effect of hypoxia. We added in our limitations that “ The concurrent effects of physical fatigue and hypoxia cannot be fully disentangled in the present design, while this reflects ecologically valid conditions for athletes, future studies are required to clarify the causal inference regarding the independent role of hypoxia on cognitive performance.”*

Page 17, Lines 238-241: The failure to capture HR and SpO2 data during the jump task removes the primary objective markers of internal physiological load. Clarifying the absence of alternative sensor placements (e.g., chest strap HR monitors) would elucidate how the missing physiological data impacts the interpretation of the physical task's fatiguing effect.

*Thank you for this comment. As stated in the manuscript (Lines 119 and 165-170 and 403-405), and indeed the goal of the jump task placed before cognitive testing was “to enhance i) the comparability with prior work employing exercise-induced fatigue prior to cognitive testing [15,20,22]), and ii) the ecological validity of the protocol and mimic mid-training cognitive demands during moguls training sessions [23,24]. This reflects real-world training scenarios where a certain amount of physical stress is concomitant to cognitive processing, such as aerial preparation or course navigation, thereby increasing the protocol’s representativeness for assessing cognitive fatigability in applied contexts.”*

*We considered the WristOx2 3150 Nonin sensors were sufficient in detecting SpO2 and HR, and we did not plan to implement cheststrap HR monitor. Pilot were not performed on moguls athletes, but by researchers, who jumped a) lower and b) with a lower frequency, and possibly not swinging hands. We will keep cheststrap this into consideration for future studies.*

Page 18, Lines 287-289: Baseline cMSIT response time was significantly worse in normoxia compared to hypoxia. The divergence in baseline performance across sessions suggests a temporal artifact or learning effect rather than mere "daily variations." Integrating temporal control designs would identify whether such baseline shifts are biological anomalies or systematic measurement artifacts [Dhahbi W, Dergaa I: Machine learning with temporal control designs for testing rhythmic specificity in chronobiology: A multivariate framework proposal for distinguishing genuine biological rhythms from temporal artifacts. Chronobiology International 2026:1-8].

*Thank you for your comment, we covariated for time of the day and we want to underline that procedures were similar and days were organized similarly across sections, with similar passation time for the athletes. We also did our best to account for any possible variation attributable to the fact that the tests were performed in different days. These limitations are acknowledged in the revised manuscript in the limitation section. Controlling by time of the day, cMSIT response time (all p<0.02) changed slightly. We adjusted the estimates. Result direction did not change. The statistical analysis section (Lines 257-259 “We adjusted for hour of testing our estimates. Since it had no effect on PVT, cMSIT accuracy or jump performance (all p≥0.10), this term was removed from the model (Occam razor principle). However, it was kept for cMSIT response time (all p<0.02).”) and the result section (Lines 296–300) have been modified accordingly. It changed also the estimated difference from hypoxia and normoxia. Now hypoxia was estimated to be ~10% worst than normoxia. This result has been adjusted in the abstract as well. We adjusted Figure 3 according to the new estimates. Also, the discussion now reads (L386-391): “The unadjusted ~7% faster response time in hypoxia would reflect pre-existing inter-session baseline differences rather than a true performance advantage. When controlling for baseline cMSIT performance, hypoxia was associated with ~10% slower performance, indicating that the covariate adjustment was essential to isolate the true condition effect from session-to-session variability, particularly in elite youth athletes whose cognitive performance may fluctuate with training and academic load.”*


Page 19, Lines 314-324 & Table 1: Reporting mean $\pm$ SD for PVT lapses ($1.5 \pm 4.25$) is statistically inappropriate for highly skewed count data. Presenting median and interquartile ranges (IQR) for all lapse data in Table 1 would accurately reflect the non-normal distribution.

*We agree with the reviewer and indeed data for Lapses in Table 1 are presented as median and interquartile range for PVT lapses. We now better flag the difference between Response time and Lapses in the notes of table 1.*

Page 20, Lines 330-332: The condition $\times$ set interaction for the jump task is marginal ($p=0.048$) with $n=14$. Reporting the confidence intervals for the post-hoc comparisons would demonstrate the precision of the finding at the third series.

*Thank you for your comment, confidence intervals for the post-hoc comparisons have been added at Lines 534–540 and in the OSF code, which now present all 95%CI for all post-hoc comparisons.*

Page 21, Lines 354-361: The discussion of SpO2 differences between simulated normobaric hypoxia and real-world hypobaric hypoxia is superficial. Contextualizing how the physiological responses to normobaric hypoxia specifically mimic or diverge from the hypobaric conditions these winter athletes actually face on the mountain would strengthen the external validity.

*Thank you for your comment, we added to the discussion more mechanistic differences between normobaric and hypobaric hypoxia at Lines 331–337.*

Reviewer #2:

We would like to thank the reviewer for his work and his observations that allowed us to improve our manuscript. We addressed all reviewers’ comments, and all changes to the manuscript are marked in red.

Abstract
The abstract presents a clear and relevant objective, addressing the assessment of cognitive fatigability in field-applied contexts, which represents a strength with practical relevance for athlete training and monitoring. However, the designation “elite youth athletes” warrants some caution, given that the sample has a mean age of 18±2 years; in this regard, the use of the term “late-adolescent elite athletes” would be more precise and scientifically rigorous.

*Thank you for your comment, we respectfully decided to keep elite youth athletes in the abstract for 3 main reasons. The first one is that late-adolescent elite athletes is not a term we found to be used in the literature, that uses mainly youth or young elite athletes. Second, we prefer to keep it more “vague” as there is already a large debate on whether it is appropriate to separate aging in discrete categories (e.g. terms such as “early adulthood”, “late adolescent”, “older adults”, “very old adults”) or if we should rather consider it as a continuum, also considering that, in terms of sport organization, elite sport is divided in youth (for most winter sports U21), and adults (world cup teams, Olympic teams). Third, we feel that indicating the age might be sufficient to describe the population. We however used this term in the introduction (see comment below).*

Introduction
The introduction presents a globally solid and well-structured conceptual foundation, guiding the reader from the general context of altitude exposure to the specific issue of cognitive fatigability. The relevance of the topic is clearly established, particularly in the context of winter sports and the increasing intermittent exposure to hypoxia. The distinction between fatigue and fatigability is a strength, demonstrating conceptual rigor and alignment with contemporary literature.
However, I believe it could benefit from some improvements:
1) Although the literature review is comprehensive, the narrative becomes somewhat lengthy and, at times, overly descriptive, particularly in the section addressing the analogy with neuromuscular fatigability. While relevant, this part could be more concise and more directly linked to the cognitive domain, avoiding some dispersion of the main focus.

*Thank you for the suggestion. We agree that this part might be dispersive and we made this part more concise (Lines 83–84). Particularly regarding the point evidenced by the reviewer, the manuscript now reads simply “Fatigability can be quantified continuously as the performance decline during a given task, or as the punctual difference in performance at a physical or cognitive test performed before, during, and after a task [6,8]. The more this test can measure the maximal capacity of a system, the more sensitive it is to detect performance impairments. Such impairments might be missed if testing is done at a submaximal level, where the sensitivity to detect force loss is lower.” As the reviewer suggested, this makes a better successive link with the part on cognitive domain.*

2) The justification of the scientific gap could be more incisive. The manuscript correctly highlights the scarcity of studies on cognitive fatigability under hypoxia and the limited transferability of laboratory protocols, but the link between these limitations and the specific need for short, field-applicable protocols could be articulated more directly and critically. In other words, a clearer transition between “what exists” and “why it is insufficient” is needed.

*Thank you. We addressed the point made by the reviewer at Lines 114–116, the paragraph has been re-worked to be more direct and concise: “While valid, the field applicability of current protocols is limited by their long duration and laboratory format, highlighting the need for short, validated tests in hypoxic conditions. Moreover, interindividual variability in terms of fitness and cognitive profiles could act as an important confounder, masking hypoxia effects.”*

3) The target population is only introduced in the final part of the introduction, without sufficiently strong prior framing. The reference to “elite youth athletes” appears somewhat late and without critical discussion. As noted in the abstract, considering that the sample has a mean age of 18±2 years, it would be more accurate to use the term “late-adolescent elite athletes,” which would also better align the sample characterization with the literature on development and sports performance.

*Thank you for your suggestion. While, for the reasons abovementioned, we prefer the term elite youth athlete, we recognize that mentioning late-adolescent elite athletes here is appropriate, also to avoid repetition at Line 118. Thus, Lines 117–118 reads: “ the current study aims to evaluate cognitive fatigability in normoxia vs. hypoxia in late-adolescent elite Canadian ski mogul athletes, a lowlander population…”*

4) Another point to consider concerns the coherence between the introduction and the study objective. Although the rationale for using short, portable tasks is present, it could be more clearly emphasized as a central element of the study’s innovation, rather than appearing diluted throughout the text.

*We address this issue at Lines 111–114. We agree that the message is presented at different points, being diluted and not strengthened. Following the reviewers suggestion, we emphasized the study innovative aspects by reducing this dilution and focusing the rationale at Lines 111–116: “While valid, the field applicability of current protocols is limited by their long duration and laboratory format, highlighting the need for short, validated tests in hypoxic conditions. Moreover, interindividual variability in terms of fitness and cognitive profiles could act as an important confounder, masking hypoxia effects. Currently, there is a need for improvements in transferability of these measures to the field (e.g. [19]). Using short, portable tasks would allow for valid and repeated assessments of cognitive fatigability in training conditions, addressing the growing need for appropriate daily monitoring in the field.”*

5) Finally, the hypothesis is clearly stated but could benefit from stronger direct grounding in the previously cited studies, thereby reinforcing its predictive rationale.

*Thank you for your suggestion. We modified the aim and hypothesis part accordingly (Lines 120–126): “We wanted to evaluate the ability of short (~15 min) protocols to detect fatigability in this population, addressing current gaps in field-deployable measures by adopting tasks with limited margin for strategy optimization (learning effect), culturally neutral and performed using an open-source touchscreen app we developed. Not allowing inter-trial recovery during the cognitive task, we hypothesized the presence of objective fatigability in normoxia as opposed to De Watcher et al. [13]. By deploying the task in hypoxia and simulating a mid-training situation, we expect fatigability to be exacerbated under hypoxia, similarly to Ochi et al. [11].”*

Methods
The methods section is, overall, detailed, transparent, and well structured, allowing for a clear understanding of the experimental procedures and supporting the reproducibility of the study. Nevertheless, I identified several aspects that warrant reflection and potential improvement:
1) The attempt to blind participants is interesting and well justified (simulation of altitude in both conditions), but it raises some concerns regarding its actual effectiveness, particularly in athletes who may be familiar with sensations associated with hypoxia. It would be useful to clarify whether participants’ perception of the experimental condition was assessed.

*The reviewer raises an important concern. To ensure blinding, we opted to make athletes naïve of the whole experimental conditions. This decision resulted of course in the inability to make a manipulation check. That is, we told athletes that both conditions were at altitude. However, asking participants if they believed to be in altitude would automatically indicate that one of the conditions was not in hypoxia. Although unconventional, we opted for not asking. We also pragmatically judged not appropriate to ask participant’s perception after the second session as i) participants entered the hypoxic room one at time, so we wanted to avoid possible cross-contamination (participants that finishes the protocol communicating about the experimental design with participants that were not tested yet); ii) we were concerned by possible expectation bias or recall bias asking perception of the experimental condition just at the end of the experiment (e.g. Allocco et al. 2025, Bial et al. 2025).*

*Participants never took part in a similar experiment and never entered a hypoxic room (we asked at the moment of inclusion). This has been now added in the methods following exclusion criteria (137-139) “None of the athletes took part in previous studies evaluating the effects of hypoxia, and none of our participants entered in a hypoxic chamber before.”*

2) Regarding the cognitive tasks, the choice of the cMSIT and PVT is well grounded and aligned with the literature. However, the operational definition of “cognitive fatigability” as the change in PVT performance pre–post cMSIT could be better conceptually justified, reinforcing the distinction between the inducing task and the outcome measure. Furthermore, the relatively short duration of the cMSIT (10 min) raises the question of whether it is sufficient to consistently induce fatigability, especially considering that previous studies cited by the authors used longer tasks.

*Thank you for your important comment and we thank the reviewer for his/her kind words. As detailed in the introduction “fatigability refers as the rate of decrement in an objective performance criterion at a given task [6].” (Lines 76-77) Thus, practically speaking, as reaction time or accuracy decrement along the task. However, pacing is an important caveat when using only this definition of fatigability. Pacing has to be considered in the study of fatigability and endurance, and similarly to most research teams we prefer to use a secondary criterion task to performed before and after the task to evaluate fatigability.(as we mentioned in Lines 81–82).
We tried to be as clear as possible in the definitions considering the endless debates on fatigue and fatigability definitions (Enoka and Duchateau 2016; Skau et al. 2021; Behrens et al. 2023; Meixner et al. 2025).
We further underlined the criterion for fatigability in the method (Lines 164–165):”* *The cognitive fatigability test consisted of 3 minutes of PVT performed before and after 10 minutes of cMSIT (Figure 1A).”*

*The rationale for using the PVT can also be found in the introduction (Lines 92–95) “Due to its short duration (i.e. 3 min. for the short version), PVT can be performed before and after the fatiguing task to provide critical validation of cognitive fatigability, ruling out the inhibitory control task-specific learning effect, ceiling effect, pacing and changes in execution strategy, which are classical confounding factors [17].”
Importantly, placing it in the introduction after the parallel between physical and cognitive fatigability measurements allow us to underline that we do not argue that PVT can be considered as the cognitive counterpart of MVIC, as it is impossible to compare the two outcomes’ construct validity. In the neuromuscular system, it is well accepted that maximal force is a surrogate of the maximal capacity of the system. We are not aware of a similar outcome that can be easily obtain as surrogate of the maximal capacity of the neural circuitry regulating important executive functions such as inhibitory control. However, PVT has been used for other interesting advantages: less salient cognitive functions are engaged (compared to inhibitory control tasks or working memory tasks), it is validated for short durations (3 min), the learning effect is absent, it is sensible to cognitive decline due to fatigue or lack of sleep (Basner 2011 a,b; 2017).*

*The reviewer also raises an important concern: the duration of the 10 min. Reducing the task duration from 30 to 10 min. was the first innovative aspect of this study, as we shown that cMSIT induced reliably fatigue after 30 min in a similar population (normoxia) but few studies adopted a 10 min duration to induce fatigue. Short duration are usually adopted for testing cognitive function (e.g. in hypoxia the study of Ochi used a ~6 min Stroop task, and the study of O’Keffe a 16 min TloadDback) but not for inducing fatigue. A 10 min. stroop task was showed to induce “mental fatigue” before, but this was a self-reported measure (Dallaway et al. 2022).*

*Previously, PVT impairments were also observed following 20-min dual task (inhibitory task + cycling) at different inhibitory control task, including the multisource interference task (Varesco et al. 2025 IJSPP a; supplmental materials).*

*For those reasons, the first aim (the first hypothesis) was indeed to verify fatigability in normoxia (Lines 201–202).*

*This point is also addressed in the limitation and future perspective, where we stated that “Future studies should evaluate the dose-response effect of task duration at different altitudes” considering that we observed increased fatigability only in hypoxia.*

3) The within-subject design is appropriate, and the selected tasks (cMSIT and PVT) are consistent with the literature for assessing inhibitory control and vigilance. However, some methodological limitations should be considered. In particular, the use of a fixed order of conditions (normoxia followed by hypoxia) may introduce learning, fatigue, or expectation effects, thereby compromising the internal validity of the findings.

*Thank you for your comment, these limitations have been acknowledged at Lines 437–445: “The main limitation of the present study is that sessions were not randomized: timely switching between conditions (a prerogative of randomization) was not possible as the hypoxic chamber requires hours to stabilize at new altitudes. To reduce the order effect, we used a sham condition (200 m) and made the athletes naïve of the study true objective. Non randomization could influence baseline cMSIT performance. We were aware of the possible learning, fatigue, or expectation effects before the study and adopted all available procedures to adress this limitation, such as performing an extensive familiarisation in both sessions, using tasks with limited learning effect and contolling for baseline cognitive performance. However, the potential impact of this limitation on the internal validity of the findings cannot be completely ruled out.”*

Results
The results section is overall detailed and technically robust, with a careful description of the statistical analyses and a clear effort to ensure the adequacy of the models used. Nevertheless, several aspects could be improved to enhance clarity, coherence, and interpretation of the findings:
1. The section is, at times, overly technical and dense, with a level of statistical detail that may compromise readability. The inclusion of extensive information on model adjustments, distributions, and assumption checks (e.g., Q-Q plots, robust models) is relevant, but could be partially moved to supplementary material, allowing for a smoother presentation of the main results.

*Thank you for your comment. We re-worked on the result to facilitate the reading. However, we deem some details necessary to justify some statistical approach implemented and corroborate the robustness of our analysis. For example, skewed distribution for response time at PVT (or other reaction time tests) are a common issue, and we deem necessary to acknowledge the presence of skewness and how we ensured robustness of our analysis (applying indeed robust LMM or GLMM). Similarly, we now tried to keep details about model adjustments minimal, however we think it is important to acknowledge the difference between the results obtained without consider baseline and when considering baseline (as did O’Keffe et al.2021).*

2. The presentation of the cMSIT results raises some issues of interpretative coherence. Initially, performance is reported as better in hypoxia (~8%), but after baseline adjustment it appears worse (~6%). While this inversion is methodologically explainable, it is not sufficiently clarified in the text and may confuse the reader. It would be important to more explicitly explain the role of baseline in this shift in interpretation.

*The apparent inversion reflects a baseline imbalance between conditions: participants had faster pre-task RT in hypoxia than normoxia sessions. Unadjusted means therefore favor hypoxia artifactually. The adjusted model thus provides the more valid estimate of condition effect. We have clarified this in the Discussion (please note that another reviewer appropriately suggested to control for time of the day in the model, so estimate changed and now performance ~7% was better in hypoxia, and 10% better in normoxia after baseline adjustment) at lines 386-391:”The unadjusted ~7% faster response time in hypoxia would reflect pre-existing inter-session baseline differences rather than a true performance advantage. When controlling for baseline cMSIT performance, hypoxia was associated with ~10% slower performance, indicating that the covariate adjustment was essential to isolate the true condition effect from session-to-session variability, particularly in elite youth athletes whose cognitive performance may fluctuate with training and academic load.”*

3. The absence of a time effect in the cMSIT (i.e., no decline over the 10 minutes) raises questions about the effectiveness of the task as an inducer of cognitive fatigability. Although the authors refer to supplementary material, this point is central to the interpretation of the results and should be more explicitly addressed in this section or, at least, acknowledged as a potential limitation.

*We agree with the reviewer on the need of more details, however, as abovementioned, we think that pacing is an important caveat in using response time at the cMSIT as index of fatigability. Pacing is inherent in virtually any task that requires protracted effort (e.g. Tucker BJSM 2009), otherwise any physical task would look like a critical power test profile. Cognitive task would present pacing as well, because they combine effort, motivation and expectation (Pageaux and Lepers, Progress in Brain Research 2018).*

*We acknowledge this point in the discussion (Lines 399–401): “response time at the cMSIT should be interpreted with caution as an index of fatigability, as pacing is inherent to any task requiring protracted effort and was possibly present in our cognitive tasks as well [35].”*

4. Regarding the assessment of fatigability through the PVT, the results are more consistent and aligned with the hypothesis, showing an effect of hypoxia. However, the magnitude of the effects (e.g., relatively small η²p values) is not discussed, which makes it difficult to assess their practical relevance. Additionally, the need to use robust models due to the presence of extreme values suggests some variability in the data that could be better contextualized.

*Thank you for your observation, we have added a part in the discussion (Lines 356–361): “The small effect size observed should not be dismissed as practically irrelevant: in elite youth athletes, even marginal impairments in psychomotor vigilance might have meaningful implications for decision-making, technical execution, and safety. Moreover, effect sizes in within-subject designs with small samples are typically conservative estimates. Finally, elite athletes likely operate at the upper end of cognitive endurance capacity, potentially making them less susceptible to fatigability induced by the cMSIT, which might be more pronounced in less fit or untrained individuals.”*

*Regarding the use of robust models, extreme values in PVT data are well-documented in the literature and do not reflect poor data quality but rather the inherent lapse-driven distribution of vigilance performance.*

5. Another important aspect concerns the loss of participants at different stages (missed sessions, exclusion from the jump task), which further reduces the sample size in some analyses. This issue is not addressed in the section and may have implications for the robustness of the findings.

*We thank the reviewer for raising this concern. Sample size loss was most pronounced in the jump task (n=14). While we acknowledge this could be seen as problematic, several points support the robustness of our findings: (1) we use linear mixed models, which handle unbalanced designs by using all available observations without listwise deletion (an advantage over other LM such as ANOVA); (2) we have added 95% CIs to post-hoc comparisons for the jump task (Lines 331–337) to transparently communicate estimation precision given the reduced sample; and (3) the pattern of results is consistent across outcomes despite varying sample sizes.*

Discussion
The discussion section shows good alignment with the study objectives and demonstrates a consistent effort to interpret the findings in light of the existing literature. Nevertheless, several aspects could be improved to strengthen the clarity, consistency, and scientific rigor of the discussion:
1. The interpretation of the cMSIT results remains somewhat ambiguous and, at times, speculative. The authors correctly acknowledge the absence of a time×condition interaction, but propose multiple possible explanations (insufficient task duration, differences in effort, inter-session variability) without clearly prioritizing the most plausible one. This multiplicity of interpretations, without a more assertive position, may weaken the main message. Moreover, the explanation based on baseline differences is relevant but could be presented more clearly and better integrated with the results.

*Thank you for your comment, we reviewed this part of the discussion (Lines 382–393):*

*“We rather propose that this is due to possible inter-session differences that were present already at baseline: athletes reported to the two experimental sessions with different baseline performance at the cMSIT, which was not observed in PVT response times. This could be expected as cMSIT reflect distinct neural processes involved in inhibitory control and choice beyond reaction time paradigms [36]. Indeed, PVT pre-cMSIT was also similar across sessions, and different immediately after. The unadjusted ~7% faster response time in hypoxia would reflect pre-existing inter-session baseline differences rather than a true performance advantage. When controlling for baseline cMSIT performance, hypoxia was associated with ~10% slower performance, indicating that the covariate adjustment was essential to isolate the true condition effect from session-to-session variability, particularly in elite youth athletes whose cognitive performance may fluctuate with training and academic load. Daily variations in cognitive function are common, considering the multitude of possible influencing factors such as sleep, nutrition, macrocycle training load, or other social and academic stressors independent of hypoxia-induced impairments [13].”*

2. Although the authors acknowledge that the 10-minute task may not have been sufficient to induce fatigability in normoxia, this limitation is central and should be more strongly emphasized. Indeed, it raises a fundamental question about the validity of the protocol for assessing cognitive fatigability, particularly considering that the operational definition of the construct depends on inducing performance decline over time.

*We acknowledge the need of more studies investigating the dose-effect properties of cognitive task at different altitudes (including normoxia) at Lines 454–456. Furthermore, we emphasized that (Lines 399–401) ”response time at the cMSIT should be interpreted with caution as an index of fatigability, as pacing is inherent to any task requiring protracted effort and was possibly present in our cognitive tasks as well [35].”*

3. Another critical point concerns the interpretative complexity introduced by including a physical task prior to cognitive assessment. While well justified from an ecological perspective, this choice makes it more difficult to disentangle the effects of hypoxia from those of physical fatigue. This limitation is only partially acknowledged and should be discussed more explicitly.

*Thank you, this has been added at Lines 351–354: “The concurrent effects of physical fatigue and hypoxia cannot be fully disentangled in the present design, while this reflects ecologically valid conditions for athletes, future studies are required to clarify the causal inference regarding the independent role of hypoxia on cognitive performance.”*

4. Regarding the comparison with the literature, it is adequate but could be more critical. For example, differences in oxygen saturation levels are mentioned as an explanation for inconsistent findings, but other relevant variables (e.g., task duration, sample characteristics) could be more systematically integrated.

*Thank you for this comment, we better discussed the specificities of the duration and our sample characteristics relative to the outcome observed (in particular when discussing about the meaningfulness of the effect size observed) at Lines 352–361 “The short duration of the task in the present study (i.e. 10 min) resulted similar PVT performance from pre- to post-cMSIT in normoxia. Only during the hypoxic condition, we observed an increase in PVT lapses and response time following the cMSIT, indicating an increased fatigability in hypoxia in line with previous data showing an increase in strain on brain regions like PFC and dACC with hypoxemia [22,31,32]. The small effect size observed should not be dismissed as practically irrelevant: in elite youth athletes, even marginal impairments in psychomotor vigilance might have meaningful implications for decision-making, technical execution, and safety. Moreover, effect sizes in within-subject designs with small samples are typically conservative estimates. Finally, elite athletes likely operate at the upper end of cognitive endurance capacity, potentially making them less susceptible to fatigability induced by the cMSIT, which might be more pronounced in less fit or untrained individuals”*

5. The limitations section is relevant and appropriately acknowledges the lack of randomization. However, the potential impact of this limitation on the internal validity of the findings could be discussed in greater depth, particularly in relation to the order effects already evidenced in the results (baseline differences).

*Following the reviewer’s comments, we worked on the limitation section, which now reads (Lines 437–456): “The main limitation of the present study is that sessions were not randomized: timely switching between conditions (a prerogative of randomization) was not possible as the hypoxic chamber requires hours to stabilize at new altitudes. To reduce the order effect, we used a sham condition (200 m) and made the athletes naïve of the study true objective. Non randomization could influence baseline cMSIT performance. We were aware of the possible learning, fatigue, or expectation effects before the study and adopted all available procedures to address this limitation, such as performing an extensive familiarisation in both sessions, using tasks with limited learning effect and controlling for baseline cognitive performance. However, the potential impact of this limitation on the internal validity of the findings cannot be completely ruled out. While the sample size was small, it comprised a large team of elite youth athletes training and competing together, minimizing inter-subject variability from sport or load differences. Regarding elite youth mogul ski athletes, our sample captures most of the target population, ensuring representativeness. However, results might not apply to different disciplines, to older athletes, to highlanders or acclimatized populations. Future studies should evaluate the dose-response effect of task duration at different altitudes. In the laboratory, there is also a need to evaluate the effect of cold combined to hypoxia, which is another environmental factor that could influence cognitive fatigability. The concurrent effects of physical fatigue and hypoxia cannot be fully disentangled in the present design, while this reflects ecologically valid conditions for athletes, future studies are required to clarify the causal inference regarding the independent role of hypoxia on cognitive performance. In the field, methodological questions include changes in response time with or without gloves and googles on, which might increase athlete comfort but might affect test results.”*
