# Supplementary material for: Assessment of cognitive performance and fatigability in elite athletes: Short and portable protocols for field monitoring under hypoxia
Source: PLoS One. 2026 Jul 10;21(7):e0353673. doi: 10.1371/journal.pone.0353673 (PMC13353968; doi:10.1371/journal.pone.0353673)
Supplement: Peer review history

# Peer review: Decision Letter 0

**DOI:** 10.1371/journal.pone.0353673.r001

**Authors:** Julio Alejandro Henriques da Costa

15 Apr 2026

PONE-D-26-11054

Objective assessment of cognitive fatigability in elite youth athletes: short protocols detect hypoxia effects for field applications

PLOS One

Dear Dr. Varesco,

Thank you for submitting your manuscript to PLOS ONE. After careful consideration, we feel that it has merit but does not fully meet PLOS ONE’s publication criteria as it currently stands. Therefore, we invite you to submit a revised version of the manuscript that addresses the points raised during the review process.

Please submit your revised manuscript by May 30 2026 11:59PM. If you will need more time than this to complete your revisions, please reply to this message or contact the journal office at plosone@plos.org. When you're ready to submit your revision, log on to https://www.editorialmanager.com/pone/ and select the 'Submissions Needing Revision' folder to locate your manuscript file.

Please include the following items when submitting your revised manuscript:

- A letter that responds to each point raised by the academic editor and reviewer(s). You should upload this letter as a separate file labeled 'Response to Reviewers'.
- A marked-up copy of your manuscript that highlights changes made to the original version. You should upload this as a separate file labeled 'Revised Manuscript with Track Changes'.
- An unmarked version of your revised paper without tracked changes. You should upload this as a separate file labeled 'Manuscript'.

If you would like to make changes to your financial disclosure, please include your updated statement in your cover letter. Guidelines for resubmitting your figure files are available below the reviewer comments at the end of this letter.

If applicable, we recommend that you deposit your laboratory protocols in protocols.io to enhance the reproducibility of your results. Protocols.io assigns your protocol its own identifier (DOI) so that it can be cited independently in the future. For instructions see: https://journals.plos.org/plosone/s/submission-guidelines#loc-laboratory-protocols. Additionally, PLOS ONE offers an option for publishing peer-reviewed Lab Protocol articles, which describe protocols hosted on protocols.io. Read more information on sharing protocols at https://plos.org/protocols?utm_medium=editorial-email&utm_source=authorletters&utm_campaign=protocols.

As the corresponding author, your ORCID iD is verified in the submission system and will appear in the published article. PLOS supports the use of ORCID, and we encourage all coauthors to register for an ORCID iD and use it as well. Please encourage your coauthors to verify their ORCID iD within the submission system before final acceptance, as unverified ORCID iDs will not appear in the published article. *Only*  the individual author can complete the verification step; PLOS staff *cannot*  verify ORCID iDs on behalf of authors.

We look forward to receiving your revised manuscript.

Kind regards,

Julio Alejandro Henriques Castro da Costa

Academic Editor

PLOS One

Journal Requirements:

When submitting your revision, we need you to address these additional requirements.

1. Please ensure that your manuscript meets PLOS ONE's style requirements, including those for file naming. The PLOS ONE style templates can be found at

https://journals.plos.org/plosone/s/file?id=wjVg/PLOSOne_formatting_sample_main_body.pdf and

https://journals.plos.org/plosone/s/file?id=ba62/PLOSOne_formatting_sample_title_authors_affiliations.pdf

2. Thank you for stating the following financial disclosure:

"This study was supported by the Ministère de l’Education du Quebec through the “Programme de soutien au développement de l'excellence (PSDE)” and was part of the synergic project “QueFreSkiNO”. We received further financial support from the PRIDI program from the National Institute of Sport of Quebec (PRIDI-76: PRIDI-95) and from the Research Center of the CIUSSS NIM. GV was supported by a Mitacs Accelerate Fellowship (IT42957). GS was supported by the FRQS Research Scholar Program (#297725)."

Please state what role the funders took in the study.  If the funders had no role, please state: "The funders had no role in study design, data collection and analysis, decision to publish, or preparation of the manuscript."

If this statement is not correct you must amend it as needed.

Please include this amended Role of Funder statement in your cover letter; we will change the online submission form on your behalf.

3. We note that you have indicated that there are restrictions to data sharing for this study. PLOS only allows data to be available upon request if there are legal or ethical restrictions on sharing data publicly. For more information on unacceptable data access restrictions, please see http://journals.plos.org/plosone/s/data-availability#loc-unacceptable-data-access-restrictions.

Before we proceed with your manuscript, please address the following prompts:

a) If there are ethical or legal restrictions on sharing a de-identified data set, please explain them in detail (e.g., data contain potentially identifying or sensitive patient information, data are owned by a third-party organization, etc.) and who has imposed them (e.g., a Research Ethics Committee or Institutional Review Board, etc.). Please also provide contact information for a data access committee, ethics committee, or other institutional body to which data requests may be sent.

b) If there are no restrictions, please upload the minimal anonymized data set necessary to replicate your study findings to a stable, public repository and provide us with the relevant URLs, DOIs, or accession numbers. For a list of recommended repositories, please see

https://journals.plos.org/plosone/s/recommended-repositories. You also have the option of uploading the data as Supporting Information files, but we would recommend depositing data directly to a data repository if possible.

We will update your Data Availability statement on your behalf to reflect the information you provide.

4. Please include captions for your Supporting Information files at the end of your manuscript, and update any in-text citations to match accordingly. Please see our Supporting Information guidelines for more information: http://journals.plos.org/plosone/s/supporting-information.

5. If the reviewer comments include a recommendation to cite specific previously published works, please review and evaluate these publications to determine whether they are relevant and should be cited. There is no requirement to cite these works unless the editor has indicated otherwise.

[Note: HTML markup is below. Please do not edit.]

Reviewers' comments:

Reviewer's Responses to Questions

**Comments to the Author**

1. Is the manuscript technically sound, and do the data support the conclusions?

The manuscript must describe a technically sound piece of scientific research with data that supports the conclusions. Experiments must have been conducted rigorously, with appropriate controls, replication, and sample sizes. The conclusions must be drawn appropriately based on the data presented.

Reviewer #1: Partly

Reviewer #2: Partly

**********

2. Has the statistical analysis been performed appropriately and rigorously?

Reviewer #1: Yes

Reviewer #2: Yes

**********

3. Have the authors made all data underlying the findings in their manuscript fully available?

The PLOS Data policy requires authors to make all data underlying the findings described in their manuscript fully available without restriction, with rare exception (please refer to the Data Availability Statement in the manuscript PDF file). The data should be provided as part of the manuscript or its supporting information, or deposited to a public repository. For example, in addition to summary statistics, the data points behind means, medians and variance measures should be available. If there are restrictions on publicly sharing data—e.g. participant privacy or use of data from a third party—those must be specified.

Reviewer #1: No

Reviewer #2: Yes

**********

4. Is the manuscript presented in an intelligible fashion and written in standard English?

PLOS ONE does not copyedit accepted manuscripts, so the language in submitted articles must be clear, correct, and unambiguous. Any typographical or grammatical errors should be corrected at revision, so please note any specific errors here.

Reviewer #1: Yes

Reviewer #2: Yes

**********

5. Review Comments to the Author

Please use the space provided to explain your answers to the questions above. You may also include additional comments for the author, including concerns about dual publication, research ethics, or publication ethics. (Please upload your review as an attachment if it exceeds 20,000 characters)

Reviewer #1: General Comments

The manuscript investigates the application of a short cognitive and physical testing protocol to assess cognitive fatigability under normobaric hypoxia in elite youth athletes. While the objective aligns with current needs in sports science for field-deployable monitoring tools, the study design and execution present significant methodological challenges that compromise the integrity of the findings.

Major Weaknesses:

Fixed-Order Crossover Design: The most critical flaw is the lack of randomization in the condition sequence. All participants completed the normoxia session first, followed by the hypoxia session two weeks later. In cognitive assessments involving inhibitory control and psychomotor vigilance, learning, practice, and habituation effects are substantial. Consequently, the effect of hypoxia is inherently confounded by the sequence effect. The baseline differences observed in cMSIT performance strongly indicate a practice effect from session 1 to session 2.

Missing Data and Sample Size: The initial sample size is small ($n=17$), which is typical for elite populations, but subject attrition reduces the analytical power further. Missing physiological data (heart rate and SpO2 during the jump task) due to equipment issues represents a significant loss of objective internal load metrics required to validate the physiological strain of the task.

App Validation: The study relies on a custom-developed iOS application for cMSIT and PVT. Peer-reviewed validation of this specific application against established gold standards (e.g., PC-based E-Prime or standard PVT-192 hardware) is absent from the manuscript.

Minor Weaknesses:

Blinding Efficacy: The authors state participants were naïve to the condition. However, symptoms of hypoxemia (SpO2 ~88%) during physical exertion would likely unblind elite athletes attuned to their physiological states. No manipulation check was reported to confirm if blinding was successful.

Statistical Reporting of Count Data: Table 1 reports count data (lapses) with means and standard deviations, despite the authors explicitly acknowledging a right-skewed Poisson distribution.

Specific Comments

Page 9, Lines 100-101: The title asserts that the protocols "detect hypoxia effects," which is a deterministic claim given the fixed-order design. Reframing the title to reflect the exploratory nature of the study would mitigate definitive claims regarding hypoxia detection.

Page 14, Lines 132-144: A formal a priori power analysis or sample size justification is absent. Providing an a priori statistical justification would determine if the study was adequately powered to detect condition $\times$ time interactions amid high inter-individual variability.

Page 14, Lines 147-148: A testing window spanning three and a half hours introduces potential circadian variation in cognitive fatigability. Controlling for exact time-of-day testing would isolate the hypoxic effect from circadian cognitive fluctuations [Amor SB, Dhahbi W, Bougrine H, Bessifi M, Geantă VA, Ursu VE, Trabelsi K, Souissi N: Differential Time-of-Day Effects of Caffeine Capsule and Mouth Rinse on Cognitive Performance in Adolescent Male Volleyball Athletes: A Randomized Crossover Investigation. Life 2025, 16(1):33].

Page 14, Lines 171-173: The protocol relies on a sham altitude (200m) to maintain blinding. Reporting post-session surveys regarding perceived physical strain would verify the actual efficacy of this blinding strategy.

Page 15, Lines 175-179: The justification for the non-randomized design relies purely on logistical convenience (chamber stabilization time). Providing secondary statistical analyses comparing session 1 vs. session 2 learning trajectories in a control group would clarify the sequence effect.

Page 16, Lines 198-212: The custom iOS application lacks formal validation against standard hardware, and touchscreen latencies vary significantly by device. Detailing the internal latency of the iPad model would demonstrate that touchscreen polling rates do not artificially skew the PVT response times. Furthermore, establishing a framework for technological validity would clarify the reliability of the derived response times [Dhahbi W, Chamari K: The Algorithmic Athlete: A Call to Standardize Assessment of Sensor Technologies and Artificial Intelligence. International Journal of Sports Physiology and Performance 2026, 1(aop):1-2].

Page 16, Lines 223-228: Utilizing repeated tuck jumps exclusively as the physical fatiguing mechanism neglects the complex multi-joint strength profiling necessary to fully assess lower-body endurance under hypoxia. Acknowledging the multi-dimensional nature of vertical jump performance would provide a more precise biomechanical context for the observed decrement in the third series [Sever O, Ceylan Hİ, Dhahbi W, Yilmaz HH, Özdemir K, Yazici AG, Jalalov F, Günay AR, Bayrakdaroğlu S, Stefanica V: Multi-joint isokinetic strength profiling as a predictor of vertical jump performance in elite freestyle wrestlers: A cross-sectional principal component analysis. Medicine 2026, 105(2):e47084].

Page 17, Lines 238-241: The failure to capture HR and SpO2 data during the jump task removes the primary objective markers of internal physiological load. Clarifying the absence of alternative sensor placements (e.g., chest strap HR monitors) would elucidate how the missing physiological data impacts the interpretation of the physical task's fatiguing effect.

Page 18, Lines 287-289: Baseline cMSIT response time was significantly worse in normoxia compared to hypoxia. The divergence in baseline performance across sessions suggests a temporal artifact or learning effect rather than mere "daily variations." Integrating temporal control designs would identify whether such baseline shifts are biological anomalies or systematic measurement artifacts [Dhahbi W, Dergaa I: Machine learning with temporal control designs for testing rhythmic specificity in chronobiology: A multivariate framework proposal for distinguishing genuine biological rhythms from temporal artifacts. Chronobiology International 2026:1-8].

Page 19, Lines 314-324 & Table 1: Reporting mean $\pm$ SD for PVT lapses ($1.5 \pm 4.25$) is statistically inappropriate for highly skewed count data. Presenting median and interquartile ranges (IQR) for all lapse data in Table 1 would accurately reflect the non-normal distribution.

Page 20, Lines 330-332: The condition $\times$ set interaction for the jump task is marginal ($p=0.048$) with $n=14$. Reporting the confidence intervals for the post-hoc comparisons would demonstrate the precision of the finding at the third series.

Page 21, Lines 354-361: The discussion of SpO2 differences between simulated normobaric hypoxia and real-world hypobaric hypoxia is superficial. Contextualizing how the physiological responses to normobaric hypoxia specifically mimic or diverge from the hypobaric conditions these winter athletes actually face on the mountain would strengthen the external validity.

Reviewer #2: Abstract

The abstract presents a clear and relevant objective, addressing the assessment of cognitive fatigability in field-applied contexts, which represents a strength with practical relevance for athlete training and monitoring. However, the designation “elite youth athletes” warrants some caution, given that the sample has a mean age of 18±2 years; in this regard, the use of the term “late-adolescent elite athletes” would be more precise and scientifically rigorous.

Introduction

The introduction presents a globally solid and well-structured conceptual foundation, guiding the reader from the general context of altitude exposure to the specific issue of cognitive fatigability. The relevance of the topic is clearly established, particularly in the context of winter sports and the increasing intermittent exposure to hypoxia. The distinction between fatigue and fatigability is a strength, demonstrating conceptual rigor and alignment with contemporary literature.

However, I believe it could benefit from some improvements:

1) Although the literature review is comprehensive, the narrative becomes somewhat lengthy and, at times, overly descriptive, particularly in the section addressing the analogy with neuromuscular fatigability. While relevant, this part could be more concise and more directly linked to the cognitive domain, avoiding some dispersion of the main focus.

2) The justification of the scientific gap could be more incisive. The manuscript correctly highlights the scarcity of studies on cognitive fatigability under hypoxia and the limited transferability of laboratory protocols, but the link between these limitations and the specific need for short, field-applicable protocols could be articulated more directly and critically. In other words, a clearer transition between “what exists” and “why it is insufficient” is needed.

3) The target population is only introduced in the final part of the introduction, without sufficiently strong prior framing. The reference to “elite youth athletes” appears somewhat late and without critical discussion. As noted in the abstract, considering that the sample has a mean age of 18±2 years, it would be more accurate to use the term “late-adolescent elite athletes,” which would also better align the sample characterization with the literature on development and sports performance.

4) Another point to consider concerns the coherence between the introduction and the study objective. Although the rationale for using short, portable tasks is present, it could be more clearly emphasized as a central element of the study’s innovation, rather than appearing diluted throughout the text.

5) Finally, the hypothesis is clearly stated but could benefit from stronger direct grounding in the previously cited studies, thereby reinforcing its predictive rationale.

Methods

The methods section is, overall, detailed, transparent, and well structured, allowing for a clear understanding of the experimental procedures and supporting the reproducibility of the study. Nevertheless, I identified several aspects that warrant reflection and potential improvement:

1) The attempt to blind participants is interesting and well justified (simulation of altitude in both conditions), but it raises some concerns regarding its actual effectiveness, particularly in athletes who may be familiar with sensations associated with hypoxia. It would be useful to clarify whether participants’ perception of the experimental condition was assessed.

2) Regarding the cognitive tasks, the choice of the cMSIT and PVT is well grounded and aligned with the literature. However, the operational definition of “cognitive fatigability” as the change in PVT performance pre–post cMSIT could be better conceptually justified, reinforcing the distinction between the inducing task and the outcome measure. Furthermore, the relatively short duration of the cMSIT (10 min) raises the question of whether it is sufficient to consistently induce fatigability, especially considering that previous studies cited by the authors used longer tasks.

3) The within-subject design is appropriate, and the selected tasks (cMSIT and PVT) are consistent with the literature for assessing inhibitory control and vigilance. However, some methodological limitations should be considered. In particular, the use of a fixed order of conditions (normoxia followed by hypoxia) may introduce learning, fatigue, or expectation effects, thereby compromising the internal validity of the findings.

Results

The results section is overall detailed and technically robust, with a careful description of the statistical analyses and a clear effort to ensure the adequacy of the models used. Nevertheless, several aspects could be improved to enhance clarity, coherence, and interpretation of the findings:

1. The section is, at times, overly technical and dense, with a level of statistical detail that may compromise readability. The inclusion of extensive information on model adjustments, distributions, and assumption checks (e.g., Q-Q plots, robust models) is relevant, but could be partially moved to supplementary material, allowing for a smoother presentation of the main results.

2. The presentation of the cMSIT results raises some issues of interpretative coherence. Initially, performance is reported as better in hypoxia (~8%), but after baseline adjustment it appears worse (~6%). While this inversion is methodologically explainable, it is not sufficiently clarified in the text and may confuse the reader. It would be important to more explicitly explain the role of baseline in this shift in interpretation.

3. The absence of a time effect in the cMSIT (i.e., no decline over the 10 minutes) raises questions about the effectiveness of the task as an inducer of cognitive fatigability. Although the authors refer to supplementary material, this point is central to the interpretation of the results and should be more explicitly addressed in this section or, at least, acknowledged as a potential limitation.

4. Regarding the assessment of fatigability through the PVT, the results are more consistent and aligned with the hypothesis, showing an effect of hypoxia. However, the magnitude of the effects (e.g., relatively small η²p values) is not discussed, which makes it difficult to assess their practical relevance. Additionally, the need to use robust models due to the presence of extreme values suggests some variability in the data that could be better contextualized.

5. Another important aspect concerns the loss of participants at different stages (missed sessions, exclusion from the jump task), which further reduces the sample size in some analyses. This issue is not addressed in the section and may have implications for the robustness of the findings.

Discussion

The discussion section shows good alignment with the study objectives and demonstrates a consistent effort to interpret the findings in light of the existing literature. Nevertheless, several aspects could be improved to strengthen the clarity, consistency, and scientific rigor of the discussion:

1. The interpretation of the cMSIT results remains somewhat ambiguous and, at times, speculative. The authors correctly acknowledge the absence of a time×condition interaction, but propose multiple possible explanations (insufficient task duration, differences in effort, inter-session variability) without clearly prioritizing the most plausible one. This multiplicity of interpretations, without a more assertive position, may weaken the main message. Moreover, the explanation based on baseline differences is relevant but could be presented more clearly and better integrated with the results.

2. Although the authors acknowledge that the 10-minute task may not have been sufficient to induce fatigability in normoxia, this limitation is central and should be more strongly emphasized. Indeed, it raises a fundamental question about the validity of the protocol for assessing cognitive fatigability, particularly considering that the operational definition of the construct depends on inducing performance decline over time.

3. Another critical point concerns the interpretative complexity introduced by including a physical task prior to cognitive assessment. While well justified from an ecological perspective, this choice makes it more difficult to disentangle the effects of hypoxia from those of physical fatigue. This limitation is only partially acknowledged and should be discussed more explicitly.

4. Regarding the comparison with the literature, it is adequate but could be more critical. For example, differences in oxygen saturation levels are mentioned as an explanation for inconsistent findings, but other relevant variables (e.g., task duration, sample characteristics) could be more systematically integrated.

5. The limitations section is relevant and appropriately acknowledges the lack of randomization. However, the potential impact of this limitation on the internal validity of the findings could be discussed in greater depth, particularly in relation to the order effects already evidenced in the results (baseline differences).

**********

6. PLOS authors have the option to publish the peer review history of their article (what does this mean?). If published, this will include your full peer review and any attached files.

If you choose “no”, your identity will remain anonymous but your review may still be made public.

**Do you want your identity to be public for this peer review?**  For information about this choice, including consent withdrawal, please see our Privacy Policy.

Reviewer #1: **Yes:**  Wissem Dhahbi

Reviewer #2: No

**********

[NOTE: If reviewer comments were submitted as an attachment file, they will be attached to this email and accessible via the submission site. Please log into your account, locate the manuscript record, and check for the action link "View Attachments". If this link does not appear, there are no attachment files.]

To ensure your figures meet our technical requirements, please review our figure guidelines: https://journals.plos.org/plosone/s/figures

You may also use PLOS’s free figure tool, NAAS, to help you prepare publication quality figures: https://journals.plos.org/plosone/s/figures#loc-tools-for-figure-preparation.

NAAS will assess whether your figures meet our technical requirements by comparing each figure against our figure specifications.

# Author comment: Author response to Decision Letter 1

**DOI:** 10.1371/journal.pone.0353673.r002

19 Apr 2026

Reviewer #1: General Comments

The manuscript investigates the application of a short cognitive and physical testing protocol to assess cognitive fatigability under normobaric hypoxia in elite youth athletes. While the objective aligns with current needs in sports science for field-deployable monitoring tools, the study design and execution present significant methodological challenges that compromise the integrity of the findings.

We would like to thank the reviewer for his kind words and his observations that allowed us to improve our manuscript. We addressed all reviewers’ comments, and all changes to the manuscript are marked in red.

Major Weaknesses:

Fixed-Order Crossover Design: The most critical flaw is the lack of randomization in the condition sequence. All participants completed the normoxia session first, followed by the hypoxia session two weeks later. In cognitive assessments involving inhibitory control and psychomotor vigilance, learning, practice, and habituation effects are substantial. Consequently, the effect of hypoxia is inherently confounded by the sequence effect. The baseline differences observed in cMSIT performance strongly indicate a practice effect from session 1 to session 2.

Thank you for this relevant comment, we acknowledge this limitation in our design, which was driven by practical needs: the hypoxic generator needs approximately 2h to ensure reliable stabilization of altitude, making it impossible to switch from low to high altitude promptly and thus making it unfeasible to divide athletes in two groups. Testing athletes on different days was also not a viable choice due to logistical constraints and, importantly, by the fact that testing athletes at different days would induce training to act as confounder; it is thus essential to test athletes from the same team the same day. This has been acknowledged at Lines 172–178.

We want to underline that PVT has been selected to test for fatigability also because it has consistently shown to not have a familiarization/learning/practice effect along a wide range of literature studies (e.g. Basner 2011). We selected the cMSIT with zero-lag between trials over other inhibitory control tasks (e.g. Stroop task, Simon task, Flanker task) as the specific properties of the task (detailed in supplemental materials S1) make the task less subject to familiarization effect. Finally, whether differences in baseline between sessions were due to familiarization, cognitive performance or the combination of both, was addressed by controlling for baseline results in our analysis. Thus, if as noted by the reviewer, there was a “baseline differences observed in cMSIT performance strongly indicate a practice effect from session 1 to session 2”, this was accounted for in our lmm.

These limitations have been acknowledged at Lines 437–445: “The main limitation of the present study is that sessions were not randomized: timely switching between conditions (a prerogative of randomization) was not possible as the hypoxic chamber requires hours to stabilize at new altitudes. To reduce the order effect, we used a sham condition (200 m) and made the athletes naïve of the study true objective. Non randomization could influence baseline cMSIT performance. We were aware of the possible learning, fatigue, or expectation effects before the study and adopted all available procedures to adress this limitation, such as performing an extensive familiarisation in both sessions, using tasks with limited learning effect and contolling for baseline cognitive performance. However, the potential impact of this limitation on the internal validity of the findings cannot be completely ruled out.”

Missing Data and Sample Size: The initial sample size is small ($n=17$), which is typical for elite populations, but subject attrition reduces the analytical power further. Missing physiological data (heart rate and SpO2 during the jump task) due to equipment issues represents a significant loss of objective internal load metrics required to validate the physiological strain of the task.

Thank you for your comment. Indeed, it is difficult to recruit large samples of elite athletes, especially from winter sports, whose teams rarely surpass the 10 members. One main advantage is that recruiting elite individuals of the same team would largely reduce possible interindividual confounders, minimizing those related to lifestyle or baseline fitness, which are a drawback in several studies evaluating active individuals (e.g. Van Cutsem et al. 2017; Martin et al. 2016]). Optimising modelling for small sample sizes is not an easy feat, but we believe our lmm could address several objections related to insufficient statistical power (e.g., Lakens, 2022). Finally, attrition was low as the totality of the participants took part in the protocol, leaving out only a couple of individuals who could not take part to testing due to influenza or injury (detailed at Lines 271–272).

We do not think that lack of SpO2 and HR data during the physical task represent a major drawback as external load was captured in the hop test (a simil-time trial / sprinting task), physiological strain should result in performance decline, as we observed in hypoxia. Furthermore, this limitation might address secondary outcomes, while the hop test goal was to mimic a mid-training situation (Lines 117–120 and 403-411).

App Validation: The study relies on a custom-developed iOS application for cMSIT and PVT. Peer-reviewed validation of this specific application against established gold standards (e.g., PC-based E-Prime or standard PVT-192 hardware) is absent from the manuscript.

We discuss this point at Lines 203–221. In a nutshell, previous app attempting to validate task using a cross-device approach failed as the device architecture was systematically different. Some adjusted the difference manually (e.g. subtracting a fix ~77 ms from the result app to improve precision; Arsintescu et al. 2019). As such, we re-validated the original construct (lapses and reaction time; Basner et al. 2011, 2017) and discussed the implication of re-adjustment vs. construct evaluation at Lines 414-423. We are convinced that this is a better approach than adjusting the sensitivity of the device to another just by re-centering a median bias.

Minor Weaknesses:

Blinding Efficacy: The authors state participants were naïve to the condition. However, symptoms of hypoxemia (SpO2 ~88%) during physical exertion would likely unblind elite athletes attuned to their physiological states. No manipulation check was reported to confirm if blinding was successful.

Thank you for your comment. To ensure blinding, we opted to make athletes naïve of the whole experimental conditions. This decision resulted of course in the inability to make a manipulation check. That is, we told athletes that both conditions were at altitude, assuming a 2 weeks recall is most likely biased, so interoceptive comparison across sessions was likely believed by the athletes themselves to be inaccurate (e.g. Allocco et al. 2025; Birol et al. 2024). That is, athletes were aware that they could not reliably compare the two sessions, and this uncertainty played in our favor, maximizing blinding when telling them that both sessions were in hypoxia (described at Lines 130-131 and 171–178 and 439-440 in the limitations).

Statistical Reporting of Count Data: Table 1 reports count data (lapses) with means and standard deviations, despite the authors explicitly acknowledging a right-skewed Poisson distribution.

We respectfully would like to point out that, as explicitly stated in the table notes, Lapses were indeed presented as median±interquartile range, not as mean±SD, a defined by the annotation “a”. , we apologize it is was not sufficiently highlighted. We have further highlighted this in the table notes in the revised version.

Specific Comments

Page 9, Lines 100-101: The title asserts that the protocols "detect hypoxia effects," which is a deterministic claim given the fixed-order design. Reframing the title to reflect the exploratory nature of the study would mitigate definitive claims regarding hypoxia detection.

Thank you for your comment, the reviewer indicated lines 100-101, which mention “Using

short, portable tasks would allow for valid and repeated assessments of cognitive fatigability in training

conditions, addressing the growing need for appropriate daily monitoring in the field.” We believe the reviewer indicates the title should better reflect this statement avoiding this deterministic claim. Thus, we modified the manuscript title in “Objective assessment of cognitive fatigability in elite youth athletes: short portable protocols for field monitoring under hypoxia”

Page 14, Lines 132-144: A formal a priori power analysis or sample size justification is absent. Providing an a priori statistical justification would determine if the study was adequately powered to detect condition $\times$ time interactions amid high inter-individual variability.

We recognize that power analysis should be population-specific.Because no prior study has examined comparable outcomes in elite youth moguls or freestyle ski athletes, an a priori power calculation based on an external effect size would have been speculative. We therefore added a sample size justification based on the rarity of the population, the feasibility of recruiting this cohort, and the repeated-measures design, which improves statistical sensitivity within participants. We prefer not to perform post hoc power analysis to prove adequacy as observed power is largely a function of the p-value and the sample effect estimate (Lakens 2022). Our within-subject repeated-measures structure (multiple assessments per participant across conditions and time points) increased statistical sensitivity and power relative to between-subject designs, even with modest sample size typical of sport science, by reducing inter-individual variability and leveraging participant-specific baselines. This approach aligns with recommendations for exploratory studies in specialized populations where population-specific effect sizes are unavailable (Lakens 2022). We have further clarified that our sample size was determined by convenience sampling and it represented the ~5% of the total population, which is large (Lines 139-141). This is further discussed in the limitations at Lines 445–449.

Page 14, Lines 147-148: A testing window spanning three and a half hours introduces potential circadian variation in cognitive fatigability. Controlling for exact time-of-day testing would isolate the hypoxic effect from circadian cognitive fluctuations [Amor SB, Dhahbi W, Bougrine H, Bessifi M, Geantă VA, Ursu VE, Trabelsi K, Souissi N: Differential Time-of-Day Effects of Caffeine Capsule and Mouth Rinse on Cognitive Performance in Adolescent Male Volleyball Athletes: A Randomized Crossover Investigation. Life 2025, 16(1):33].’

Thank you for this relevant comment. Following the reviewer’s suggestion, we adjusted for hour of testing all our outcomes. The adjustment did not influence PVT (all p>0.19), cMSIT accuracy (p=0.95) or jump performance (p=0.10) and thus removed from the model as per the Occam razor principle of parsimony, avoiding overfitting (Burnham & Anderson, 2002). However, it had an effect on cMSIT response time (all p<0.02) and thus kept in the model. While this did not influence the results, it changed slightly the estimates, overall improving the precision of the model and confirming our result.

The statistical analysis section (Lines 257-259 “We adjusted for hour of testing our estimates. Since it had no effect on PVT, cMSIT accuracy or jump performance (all p≥0.10), this term was removed from the model (Occam razor principle). However, it was kept for cMSIT response time (all p<0.02).”) and the result section (Lines 296–300) have been modified accordingly. It changed also the estimated difference from hypoxia and normoxia. Now hypoxia was estimated to be ~10% worst than normoxia. This result has been adjusted in the abstract as well. We adjusted Figure 3 according to the new estimates. Also, the discussion now reads (L386-391): “The unadjusted ~7% faster response time in hypoxia would reflect pre-existing inter-session baseline differences rather than a true performance advantage. When controlling for baseline cMSIT performance, hypoxia was associated with ~10% slower performance, indicating that the covariate adjustment was essential to isolate the true condition effect from session-to-session variability, particularly in elite youth athletes whose cognitive performance may fluctuate with training and academic load.”

Page 14, Lines 171-173: The protocol relies on a sham altitude (200m) to maintain blinding. Reporting post-session surveys regarding perceived physical strain would verify the actual efficacy of this blinding strategy.

Unfortunately, we did not record perceived physical strain. We will keep this suggestion in mind for future studies, thank you.

Page 15, Lines 175-179: The justification for the non-randomized design relies purely on logistical convenience (chamber stabilization time). Providing secondary statistical analyses comparing session 1 vs. session 2 learning trajectories in a control group would clarify the sequence effect.

We do not fully understand what should be compared to what control group as there was no such group in our study, but these limitations have been acknowledged at Lines 437–445:” The main limitation of the present study is that sessions were not randomized: timely switching between conditions (a prerogative of randomization) was not possible as the hypoxic chamber requires hours to stabilize at new altitudes. To reduce the order effect, we used a sham condition (200 m) and made the athletes naïve of the study true objective. Non randomization could influence baseline cMSIT performance. We were aware of the possible learning, fatigue, or expectation effects before the study and adopted all available procedures to adress this limitation, such as performing an extensive familiarisation in both sessions, using tasks with limited learning effect and contolling for baseline cognitive performance. However, the potential impact of this limitation on the internal validity of the findings cannot be completely ruled out.”

We want to underline that if as noted by the reviewer, there was a “baseline differences observed in cMSIT performance strongly indicate a practice effect from session 1 to session 2”, this was accounted for in our lmm.

Page 16, Lines 198-212: The custom iOS application lacks formal validation against standard hardware, and touchscreen latencies vary significantly by device. Detailing the internal latency of the iPad model would demonstrate that touchscreen polling rates do not artificially skew the PVT response times. Furthermore, establishing a framework for technological validity would clarify the reliability of the derived response times [Dhahbi W, Chamari K: The Algorithmic Athlete: A Call to Standardize Assessment of Sensor Technologies and Artificial Intelligence. International Journal of Sports Physiology and Performance 2026, 1(aop):1-2].

We added iPad model specifications and latency details (lines 221-224): “The tablet used (iPad 10th generation, 2022, A14 Bionic; iOS 18.7.7) featured a 60 Hz touchscreen polling (~16.7 ms interval) and end-to-end latency of 70-90 ms per benchmarks, which justifies critical re-evaluation of threshold for lapses that we performed following the rationale presented in Basner et al. [16] original PVT work”. Furthermore, as abovementioned, we re-validated the original construct (lapses and reaction time) and discussed the implication of re-adjustment vs. construct evaluation (L414-434).. We proceeded as the original works evaluating lapses threshold for the PVT-192 device (Basner 2011).

Page 16, Lines 223-228: Utilizing repeated tuck jumps exclusively as the physical fatiguing mechanism neglects the complex multi-joint strength profiling necessary to fully assess lower-body endurance under hypoxia. Acknowledging the multi-dimensional nature of vertical jump performance would provide a more precise biomechanical context for the observed decrement in the t

Attachment Submitted filename: Reviewer_response_R1.docx

# Peer review: Decision Letter 1

**DOI:** 10.1371/journal.pone.0353673.r003

**Authors:** Julio Alejandro Henriques da Costa, Julio Alejandro Henriques da Costa

26 Apr 2026

PONE-D-26-11054R1

Objective assessment of cognitive fatigability in elite youth athletes: short portable protocols for field monitoring under hypoxia

PLOS One

Dear Dr. Varesco,

Thank you for submitting your manuscript to PLOS ONE. After careful consideration, we feel that it has merit but does not fully meet PLOS ONE’s publication criteria as it currently stands. Therefore, we invite you to submit a revised version of the manuscript that addresses the points raised during the review process.

Please submit your revised manuscript by Jun 10 2026 11:59PM. If you will need more time than this to complete your revisions, please reply to this message or contact the journal office at plosone@plos.org. When you're ready to submit your revision, log on to https://www.editorialmanager.com/pone/ and select the 'Submissions Needing Revision' folder to locate your manuscript file.

Please include the following items when submitting your revised manuscript:

- A letter that responds to each point raised by the academic editor and reviewer(s). You should upload this letter as a separate file labeled 'Response to Reviewers'.
- A marked-up copy of your manuscript that highlights changes made to the original version. You should upload this as a separate file labeled 'Revised Manuscript with Track Changes'.
- An unmarked version of your revised paper without tracked changes. You should upload this as a separate file labeled 'Manuscript'.

If you would like to make changes to your financial disclosure, please include your updated statement in your cover letter. Guidelines for resubmitting your figure files are available below the reviewer comments at the end of this letter.

If applicable, we recommend that you deposit your laboratory protocols in protocols.io to enhance the reproducibility of your results. Protocols.io assigns your protocol its own identifier (DOI) so that it can be cited independently in the future. For instructions see: https://journals.plos.org/plosone/s/submission-guidelines#loc-laboratory-protocols. Additionally, PLOS ONE offers an option for publishing peer-reviewed Lab Protocol articles, which describe protocols hosted on protocols.io. Read more information on sharing protocols at https://plos.org/protocols?utm_medium=editorial-email&utm_source=authorletters&utm_campaign=protocols.

As the corresponding author, your ORCID iD is verified in the submission system and will appear in the published article. PLOS supports the use of ORCID, and we encourage all coauthors to register for an ORCID iD and use it as well. Please encourage your coauthors to verify their ORCID iD within the submission system before final acceptance, as unverified ORCID iDs will not appear in the published article. *Only*  the individual author can complete the verification step; PLOS staff *cannot*  verify ORCID iDs on behalf of authors.

We look forward to receiving your revised manuscript.

Kind regards,

Julio Alejandro Henriques Castro da Costa

Academic Editor

PLOS One

Journal Requirements:

If the reviewer comments include a recommendation to cite specific previously published works, please review and evaluate these publications to determine whether they are relevant and should be cited. There is no requirement to cite these works unless the editor has indicated otherwise.

Please review your reference list to ensure that it is complete and correct. If you have cited papers that have been retracted, please include the rationale for doing so in the manuscript text, or remove these references and replace them with relevant current references. Any changes to the reference list should be mentioned in the rebuttal letter that accompanies your revised manuscript. If you need to cite a retracted article, indicate the article’s retracted status in the References list and also include a citation and full reference for the retraction notice.

[Note: HTML markup is below. Please do not edit.]

Reviewers' comments:

Reviewer's Responses to Questions

**Comments to the Author**

1. If the authors have adequately addressed your comments raised in a previous round of review and you feel that this manuscript is now acceptable for publication, you may indicate that here to bypass the “Comments to the Author” section, enter your conflict of interest statement in the “Confidential to Editor” section, and submit your "Accept" recommendation.

Reviewer #1: All comments have been addressed

Reviewer #2: (No Response)

**********

2. Is the manuscript technically sound, and do the data support the conclusions?

The manuscript must describe a technically sound piece of scientific research with data that supports the conclusions. Experiments must have been conducted rigorously, with appropriate controls, replication, and sample sizes. The conclusions must be drawn appropriately based on the data presented.

Reviewer #1: Partly

Reviewer #2: Partly

**********

3. Has the statistical analysis been performed appropriately and rigorously?

Reviewer #1: Yes

Reviewer #2: Yes

**********

4. Have the authors made all data underlying the findings in their manuscript fully available?

The PLOS Data policy requires authors to make all data underlying the findings described in their manuscript fully available without restriction, with rare exception (please refer to the Data Availability Statement in the manuscript PDF file). The data should be provided as part of the manuscript or its supporting information, or deposited to a public repository. For example, in addition to summary statistics, the data points behind means, medians and variance measures should be available. If there are restrictions on publicly sharing data—e.g. participant privacy or use of data from a third party—those must be specified.

Reviewer #1: Yes

Reviewer #2: Yes

**********

5. Is the manuscript presented in an intelligible fashion and written in standard English?

PLOS ONE does not copyedit accepted manuscripts, so the language in submitted articles must be clear, correct, and unambiguous. Any typographical or grammatical errors should be corrected at revision, so please note any specific errors here.

Reviewer #1: Yes

Reviewer #2: Yes

**********

6. Review Comments to the Author

Please use the space provided to explain your answers to the questions above. You may also include additional comments for the author, including concerns about dual publication, research ethics, or publication ethics. (Please upload your review as an attachment if it exceeds 20,000 characters)

Reviewer #1: General Comments

Major Weaknesses:

Physiological Validation: The failure to collect usable heart rate and blood oxygen saturation data during the jump task due to sensor displacement limits the physiological validation of the hypoxic stimulus during the actual physical effort. Relying solely on resting data is a limitation that must be addressed more prominently.

Statistical Covariates: The rationale for replacing sex with body mass in the linear mixed-effects models requires stronger physiological justification within the main text. Sex differences in neuromuscular and cognitive fatigability are well-documented and extend beyond simple anthropometric differences.

Minor Weaknesses:

Terminology: The manuscript occasionally interchanges "hypoxia" (environmental condition) and "hypoxemia" (physiological state). Strict differentiation is required.

Formatting and Typography: There are several typographical errors, missing punctuations in statistical reporting, and inconsistent formatting in Table 1 that require correction.

Specific Comments

Page 2, Line 64: Specify the number of male athletes. You list "$n=17$, 7 women," but the male count should be explicit for immediate clarity.

Page 2, Line 82: Insert "a" before "condition set interaction" and use the standard notation "condition $\times$ set".

Page 4, Line 124: Change "refers as" to "is defined as" or "refers to".

Page 6, Line 227: There is a typographical error and a missing closing parenthesis in the Brugmann scale reporting: "physical item 3 2)". Correct the value and add the parenthesis.

Page 8, Line 282: The use of a sham condition (200 m) involves participant deception. Specify in the ethical procedures section if a debriefing occurred post-study.

Page 11, Line 396: The loss of oximetry data during the jump task is briefly mentioned. Add a sentence in the Limitations section acknowledging that the exact degree of hypoxemia during the physical task remains unquantified.

Page 11, Line 427: Adjusting for body mass instead of sex is a specific analytical choice. Add a brief sentence in this section justifying why body mass is a superior predictor for this specific youth mogul skier cohort, rather than referring solely to the supplement.

Page 12, Line 447: The phrase "influenza, different individuals" is grammatically awkward. Revise to "due to influenza (different individuals)."

Page 13, Line 475: Missing a semicolon or comma before the p-value in the accuracy data: "$(97\pm2\% \eta^{2}p=0.11 p=0.18)$".

Page 13, Line 489: Missing the subscript "$p$" for partial eta squared: "$\eta^{2}<0.001$ $p=0.98$".

Page 14, Line 521 (Table 1): The formatting is inconsistent. The response time cells for Hypoxia and Normoxia lack the standard deviation symbol (e.g., "363 74" should be "363 $\pm$ 74").

Page 16, Line 604: Change "different effort put in the task" to "differences in effort exerted during the task".

Page 18, Line 676: Specify the exact statistical threshold or method used to determine the curvilinear association mentioned.

Page 19, Line 712: Change the comma to a period or semicolon before "while this reflects ecologically valid conditions" to correct the run-on sentence.

Reviewer #2: I would like to thank the authors for their thorough and thoughtful revision of the manuscript. The responses provided demonstrate a clear effort to address the reviewers’ comments, and several aspects of the manuscript have been improved, particularly in terms of clarity, structure, and justification of methodological choices.

That said, I would like to highlight a few remaining concerns that, in my view, would benefit from further clarification and strengthening.

First, regarding the blinding procedure, although the authors provide a rationale for not including a manipulation check, the absence of any assessment of participants’ perception of the experimental condition remains a relevant limitation. Given the nature of hypoxia and the population studied, this issue should be more explicitly acknowledged in the manuscript as a potential source of bias affecting internal validity.

Second, the operationalization of cognitive fatigability, particularly the use of pre–post changes in PVT performance following the cMSIT, is better justified in the revised version. However, the conceptual distinction between the inducing task and the outcome measure could still be more clearly articulated, especially considering the broader theoretical debate around fatigability assessment.

Third, the duration of the cMSIT (10 minutes) remains a critical point. The absence of a time effect within the task raises important questions about its effectiveness as an inducer of cognitive fatigability. While this is acknowledged, I believe this limitation should be more strongly emphasized and more directly discussed in relation to the validity of the fatigability construct used in the study.

Finally, I would also encourage the authors to further reinforce the discussion around the interpretative complexity introduced by the inclusion of a physical task prior to cognitive assessment. Although this choice enhances ecological validity, it also limits the ability to disentangle the independent effects of hypoxia and physical fatigue, and this should be clearly and critically reflected in the discussion.

Addressing these points would, in my opinion, further strengthen the manuscript and improve its conceptual clarity and methodological transparency.

**********

7. PLOS authors have the option to publish the peer review history of their article (what does this mean?). If published, this will include your full peer review and any attached files.

If you choose “no”, your identity will remain anonymous but your review may still be made public.

**Do you want your identity to be public for this peer review?**  For information about this choice, including consent withdrawal, please see our Privacy Policy.

Reviewer #1: **Yes:**  Wissem Dhahbi

Reviewer #2: **Yes:**  Ana Filipa Silva

**********

[NOTE: If reviewer comments were submitted as an attachment file, they will be attached to this email and accessible via the submission site. Please log into your account, locate the manuscript record, and check for the action link "View Attachments". If this link does not appear, there are no attachment files.]

To ensure your figures meet our technical requirements, please review our figure guidelines: https://journals.plos.org/plosone/s/figures

You may also use PLOS’s free figure tool, NAAS, to help you prepare publication quality figures: https://journals.plos.org/plosone/s/figures#loc-tools-for-figure-preparation.

NAAS will assess whether your figures meet our technical requirements by comparing each figure against our figure specifications.

# Author comment: Author response to Decision Letter 2

**DOI:** 10.1371/journal.pone.0353673.r004

16 Jun 2026

Dear editor,

Thank you for letting us revise our manuscript and providing additional time to do so.

Response to all comments of the reviewers are provided in the current letter and all changes have been marked in red in the revised manuscript.

To address the comments regarding our design’s limitations, we ran a second data collection (called in the manuscript “Study II”), where we addressed the main limitations of the study, i.e. the non-randomized sessions and the inability to separate the effect of jump task effect on cognitive performance. To address these limitations and improve internal validity (i.e. running our own replication study), we collected data one year later on the same team during the pre-season period. Athletes were tested 3 weeks apart and assigned to two groups balanced for age and sex but otherwise randomized. Procedures remained similar to the previous data collection (familiarization, baseline testing, and cognitive testing), but one group performed cognitive testing in hypoxia during the first session while the other did so during the second session, in a crossover design. No jump task was performed, isolating hypoxia’s effect on cognitive performance. Athletes were tested in the same order and time across sections. The manuscript has been modified accordingly and now better support the initial findings, evidencing how the cognitive task was able to detect hypoxia-related impairments in cognitive function. We hope that we addressed all the concerns of the reviewers from the previous and current round. The revised abstract summarizes the results.

“Purpose: This study aimed to evaluate whether short (~15 min) cognitive protocols can detect fatigability in normoxia versus normobaric hypoxia in elite youth athletes, using tasks directly transferable to the field.

Methods: Elite youth mogul skiers (18±2 y) completed two studies. In Study I, 17 athletes (7 women) performed a cognitive test after a repeated tuck-jump protocol designed to mimic mid-training physical demands under two conditions in fixed order: sham normoxia (simulated 200 m) and normobaric hypoxia (3500 m, blood saturation ~88%). In Study II, 16 athletes (6 women) performed the same cognitive test without the jump task, with conditions randomized. In both studies, response time and accuracy during a 10-min color Multisource Interference Task (cMSIT) were used to assess cognitive performance, and a 3-min Psychomotor Vigilance Task (PVT) was administered before and after the cMSIT to quantify fatigability. Cognitive tests were performed on portable tablet devices.

Results: After adjusting for baseline cognitive performance, cMSIT response time was greater in hypoxia than in normoxia in both Study I (10±2%, p=0.004) and Study II (4±1%, p<0.001). Accuracy remained high and similar across conditions in both studies (Study I: 96±3%; Study II: 96±0.5%; p>0.09). In Study I, PVT response time increased only in hypoxia, from 363±74 ms to 375±89 ms (p=0.021), and lapses increased from 1±0.5 to 3±2.5 (p<0.001), whereas lapses remained unchanged in normoxia and in Study II (p>0.82). In Study I, jump task performance showed a condition×set interaction (p=0.048), with lower performance in the third set in hypoxia (19806±2464 N·s) than in normoxia (20130±3016 N·s; p=0.012).

Conclusions: In elite youth athletes, hypoxemia reduces cognitive performance and could lead to increased fatigability. Importantly, hypoxia-related impairments are detectable with short portable cognitive assessments, which are promising tools for field monitoring, particularly when tasks combines cognitive and physical demands.”

Reviewer #1: General Comments

Major Weaknesses:

Physiological Validation: The failure to collect usable heart rate and blood oxygen saturation data during the jump task due to sensor displacement limits the physiological validation of the hypoxic stimulus during the actual physical effort. Relying solely on resting data is a limitation that must be addressed more prominently.

Statistical Covariates: The rationale for replacing sex with body mass in the linear mixed-effects models requires stronger physiological justification within the main text. Sex differences in neuromuscular and cognitive fatigability are well-documented and extend beyond simple anthropometric differences.

Thank you for your comments, we addressed these points in the response to the specific comments, and we added more information on the limitation section and the statistical analysis section to address the reviewer major concerns.

Please note that, considering this round and the previous round of comments and the major limitation our design had in investigating our hypothesis, we ran a second data collection (called in the manuscript “Study II”), where we addressed the main limitations of the study, i.e. the non-randomized sessions and the inability to separate the effect of jump task effect on cognitive performance. To address these limitations and improve internal validity, we collected data one year later on the same team during the pre-season period. Athletes were tested 3 weeks apart and assigned to two groups balanced for age and sex but otherwise randomized. Procedures remained similar to the previous data collection (familiarization, baseline testing, and cognitive testing), but one group performed cognitive testing in hypoxia during the first session while the other did so during the second session, in a crossover design. No jump task was performed, isolating hypoxia’s effect on cognitive performance. Athletes were tested in the same order and time across sections. The manuscript has been modified accordingly and now better support the initial findings, evidencing how the cognitive task was able to detect hypoxia-related impairments in cognitive function. Thank you again for your comments that allowed us to improve our manuscript, we hope that we addressed all the concerns of the reviewer from the previous and current round.

Minor Weaknesses:

Terminology: The manuscript occasionally interchanges "hypoxia" (environmental condition) and "hypoxemia" (physiological state). Strict differentiation is required.

Thank you for your comment. We were very careful in using hypoxia when referring to the environmental condition imposed, and hypoxaemia when considering the physiological state driving consequences in physical and cognitive performance. We double-checked the use of hypoxia and hypoxaemia across the manuscript.

Formatting and Typography: There are several typographical errors, missing punctuations in statistical reporting, and inconsistent formatting in Table 1 that require correction.

Thank you, we completely proofread the manuscript.

Specific Comments

Page 2, Line 64: Specify the number of male athletes. You list "$n=17$, 7 women," but the male count should be explicit for immediate clarity.

We respectfully decided to keep the information as presented, if over 17 participants 7 are biological women, we prefer to avoid redundancy specifying that the remaining 10 are biological males.

Page 2, Line 82: Insert "a" before "condition set interaction" and use the standard notation "condition $\times$ set".

We apologize if we are missing a detail, but we were unable to spot a missing “×” across the manuscript.

Page 4, Line 124: Change "refers as" to "is defined as" or "refers to".

Thank you, this has been corrected.

Page 6, Line 227: There is a typographical error and a missing closing parenthesis in the Brugmann scale reporting: "physical item 3 2)". Correct the value and add the parenthesis.

We apologize if we are missing a detail but the manuscript reads:

“excessive daytime fatigue (Brugmann scale: mental item=3±1, physical item=3±2),”

Page 8, Line 282: The use of a sham condition (200 m) involves participant deception. Specify in the ethical procedures section if a debriefing occurred post-study.

Thank you for this important comment. Yes, a debrief occurred and feedback was provided. The manuscript now reads: “Due to the use of a sham condition (200 m), participants were not informed of the true altitude exposure until after data collection. All participants were debriefed post-study, and feedback regarding the study procedures and aims was provided.”

Page 11, Line 396: The loss of oximetry data during the jump task is briefly mentioned. Add a sentence in the Limitations section acknowledging that the exact degree of hypoxemia during the physical task remains unquantified.

Thank you, we added to the limitations: Due to loss of oximetry data during the jump task, the exact degree of hypoxemia during the physical task remains unquantified.

Page 11, Line 427: Adjusting for body mass instead of sex is a specific analytical choice. Add a brief sentence in this section justifying why body mass is a superior predictor for this specific youth mogul skier cohort, rather than referring solely to the supplement.

Thank you, we modified the sentence to better reflect our rationale and analyses:

Previous studies have shown that body mass (continuous variable), rather than binary sex, provides superior prediction of jump performance outcomes [30] (see Supporting Information S2 for demonstration and data divided by sex). For cognitive testing, the sex factor was removed from the models as it did not affect the results (all p>0.07).

Page 12, Line 447: The phrase "influenza, different individuals" is grammatically awkward. Revise to "due to influenza (different individuals)."

Thank you, we modified the manuscript as suggested.

Page 13, Line 475: Missing a semicolon or comma before the p-value in the accuracy data: "$(97\pm2\% \eta^{2}p=0.11 p=0.18)$".

We apologize if we are missing a detail but we were unable to spot a missing semicolon or comma. The manuscript reads: “No difference in cMSIT accuracy was found between conditions at baseline (97±2%; η2p=0.11; p=0.18).”

Page 13, Line 489: Missing the subscript "$p$" for partial eta squared: "$\eta^{2}<0.001$ $p=0.98$".

Thank you, we modified the manuscript as suggested.

Page 14, Line 521 (Table 1): The formatting is inconsistent. The response time cells for Hypoxia and Normoxia lack the standard deviation symbol (e.g., "363 74" should be "363 $\pm$ 74").

We apologize if we are missing a detail, but we were unable to spot a missing “±”. Table 1 reads “363±74”

Page 16, Line 604: Change "different effort put in the task" to "differences in effort exerted during the task".

Thank you, we modified the manuscript as suggested.

Page 18, Line 676: Specify the exact statistical threshold or method used to determine the curvilinear association mentioned.

Thank you, added the term “quadratic” to detail the type of curvilinear association that we expected.

Page 19, Line 712: Change the comma to a period or semicolon before "while this reflects ecologically valid conditions" to correct the run-on sentence.

Thank you, we modified the manuscript as suggested.

Reviewer #2: I would like to thank the authors for their thorough and thoughtful revision of the manuscript. The responses provided demonstrate a clear effort to address the reviewers’ comments, and several aspects of the manuscript have been improved, particularly in terms of clarity, structure, and justification of methodological choices.

That said, I would like to highlight a few remaining concerns that, in my view, would benefit from further clarification and strengthening.

Thank you for your comments that helped us improving our manuscript. All comments have been addressed and modifications/addition marked in red.

First, regarding the blinding procedure, although the authors provide a rationale for not including a manipulation check, the absence of any assessment of participants’ perception of the experimental condition remains a relevant limitation. Given the nature of hypoxia and the population studied, this issue should be more explicitly acknowledged in the manuscript as a potential source of bias affecting internal validity.

To address the reviewers comments regarding our design’s limitations, we ran a second data collection (called in the manuscript “Study II”), where we addressed the main limitations of the study, i.e. the non-randomized sessions and the inability to separate the effect of jump task effect on cognitive performance. To address these limitations and improve internal validity (i.e. running our own replication study), we collected data one year later on the same team during the pre-season period. Athletes were tested 3 weeks apart and assigned to two groups balanced for age and sex but otherwise randomized. Procedures remained similar to the previous data collection (familiarization, baseline testing, and cognitive testing), but one group performed cognitive testing in hypoxia during the first session while the other did so during the second session, in a crossover design. No jump task was performed, isolating hypoxia’s effect on cognitive performance. Athletes were tested in the same order and time across sections. The manuscript has been modified accordingly and now better support the initial findings, evidencing how the cognitive task was able to detect hypoxia-related impairments in cognitive function.

Second, the operationalization of cognitive fatigability, particularly the use of pre–post changes in PVT performance following the cMSIT, is better justified in the revised version. However, the conceptual distinction between the inducing task and the outcome measure could still be more clearly articulated, especially considering the broader theoretical debate around fatigability assessment.

Thank you for evidencing this important aspect. Fatigability in the present work (and in accordance with a large part of the literature) represents the decline in a given performance outcome over a specific amount of time in the case of constant-load tasks. When the load is not constant, or the effort provided is not constant (depending on the task), it becomes arguably inaccurate to assess fatigability solely on the base of performance changes over time. Thus, a common strategy is the use of a “confirmation” test performed before and after the task. The cMSIT is designed to stress inhibitory control and offers the advantage to let the athlete to “pace”, that is, change the amount of effort put into the task completion based on cognitive resources available to achieve the goal, i.e. accruing as many correct trials as possible. Combining the results of Study I and Study II, it appears that i) the presence of a physical task before the cognitive task exacerbate the effect of hypoxia in reducing cognitive performance and increasing fatigability; ii) in the presence of the physical task, individuals might be pre-fatigue, and might adopt a more conservative pacing strategy, so performance across the cMSIT did not decline over time but fatigability was observed via changes pre- to post-cMSIT in PVT performance; and iii) the cognitive task alone, without pre-fatigue, let athletes to optimize pacing strategy to achieve a better performance over time but the effect on fatigability are arguably reduced.

Third, the duration of the cMSIT (10 minutes) remains a critical point. The absence of a time effect within the task raises important questions about its effectiveness as an inducer of cognitive fatigability. While this is acknowledged, I believe this limitation should be more strongly emphasized and more directly discussed in relation to the validity of the fatigability construct used in the study.

Thank you for evidencing this important point. We now better address this issue in the discussion:

“These results also suggest that time on task was too short to induce large impairments in our experimental conditions, despite the high cognitive resources mobilized in both normoxia and hypoxia, as shown by the results of the NASA-TLX questionnaire. This is not surprising as athletes present low fatigability compared to non-athletes [40], and longer task durations might be needed. For instance, in a previous study we observed that a 30-minute cMSIT was sufficient to consistently induce impairments in their PVT performance [26]. Our results underline the need to test for different outcomes that might impact fatigability and performance

Attachment Submitted filename: Response to reviewers_R2.docx

# Peer review: Decision Letter 2

**DOI:** 10.1371/journal.pone.0353673.r005

**Authors:** Julio Alejandro Henriques da Costa, Julio Alejandro Henriques da Costa, Julio Alejandro Henriques da Costa

29 Jun 2026

Assessment of cognitive performance and fatigability in elite athletes: short and portable protocols for field monitoring under hypoxia

PONE-D-26-11054R2

Dear Dr. Varesco,

We’re pleased to inform you that your manuscript has been judged scientifically suitable for publication and will be formally accepted for publication once it meets all outstanding technical requirements.

Within one week, you’ll receive an e-mail detailing the required amendments. When these have been addressed, you’ll receive a formal acceptance letter and your manuscript will be scheduled for publication.

An invoice will be generated when your article is formally accepted. Please note, if your institution has a publishing partnership with PLOS and your article meets the relevant criteria, all or part of your publication costs will be covered. Please make sure your user information is up-to-date by logging into Editorial Manager at Editorial Manager® and clicking the ‘Update My Information' link at the top of the page. For questions related to billing, please contact billing support.

If your institution or institutions have a press office, please notify them about your upcoming paper to help maximize its impact. If they’ll be preparing press materials, please inform our press team as soon as possible -- no later than 48 hours after receiving the formal acceptance. Your manuscript will remain under strict press embargo until 2 pm Eastern Time on the date of publication. For more information, please contact onepress@plos.org.

Kind regards,

Julio Alejandro Henriques Castro da Costa

Academic Editor

PLOS One

Additional Editor Comments (optional):

Reviewers' comments:

Reviewer's Responses to Questions

**Comments to the Author**

1. If the authors have adequately addressed your comments raised in a previous round of review and you feel that this manuscript is now acceptable for publication, you may indicate that here to bypass the “Comments to the Author” section, enter your conflict of interest statement in the “Confidential to Editor” section, and submit your "Accept" recommendation.

Reviewer #1: All comments have been addressed

Reviewer #2: (No Response)

**********

2. Is the manuscript technically sound, and do the data support the conclusions?

The manuscript must describe a technically sound piece of scientific research with data that supports the conclusions. Experiments must have been conducted rigorously, with appropriate controls, replication, and sample sizes. The conclusions must be drawn appropriately based on the data presented.

Reviewer #1: Yes

Reviewer #2: Partly

**********

3. Has the statistical analysis been performed appropriately and rigorously?

Reviewer #1: Yes

Reviewer #2: Yes

**********

4. Have the authors made all data underlying the findings in their manuscript fully available?

The PLOS Data policy requires authors to make all data underlying the findings described in their manuscript fully available without restriction, with rare exception (please refer to the Data Availability Statement in the manuscript PDF file). The data should be provided as part of the manuscript or its supporting information, or deposited to a public repository. For example, in addition to summary statistics, the data points behind means, medians and variance measures should be available. If there are restrictions on publicly sharing data—e.g. participant privacy or use of data from a third party—those must be specified.

Reviewer #1: Yes

Reviewer #2: Yes

**********

5. Is the manuscript presented in an intelligible fashion and written in standard English?

PLOS ONE does not copyedit accepted manuscripts, so the language in submitted articles must be clear, correct, and unambiguous. Any typographical or grammatical errors should be corrected at revision, so please note any specific errors here.

Reviewer #1: Yes

Reviewer #2: Yes

**********

6. Review Comments to the Author

Please use the space provided to explain your answers to the questions above. You may also include additional comments for the author, including concerns about dual publication, research ethics, or publication ethics. (Please upload your review as an attachment if it exceeds 20,000 characters)

Reviewer #1: The revised manuscript (PONED-26-11054R2) evaluates the utility of short, field-deployable cognitive protocols to detect fatigability under normobaric hypoxia in elite youth mogul skiers. The authors have uniquely strengthened their original design by conducting a subsequent randomized crossover study (Study II) to isolate the environmental effects of hypoxia from pre-fatiguing physical exercise. This replication drastically improves the internal validity of the research framework. The methodological adjustment regarding touchscreen-specific PVT lapse thresholds (>500) provides essential clarity for applied sports science settings. The text is precise, the statistical treatment is sound, and the manuscript is ready for publication.

Reviewer #2: Dear Author,

please see below my comments and suggestions:

1. Construct validity of the cognitive fatigability outcome

One of my main concerns relates to the construct validity of the primary outcome, namely "cognitive fatigability". Throughout the manuscript, the authors state that the proposed protocol is able to detect cognitive fatigability under hypoxic conditions. However, I am not entirely convinced that the current experimental design fully supports this conclusion.

Cognitive fatigability is generally understood as a progressive decline in cognitive performance during sustained mental effort. In the present study, however, cognitive fatigability is inferred indirectly from changes in Psychomotor Vigilance Task (PVT) performance before and after completion of the cMSIT, rather than from a progressive deterioration in performance during the cognitive task itself. Interestingly, performance during the cMSIT itself showed little or no evidence of temporal decline, particularly in Study I.

Although the authors provide a rationale for using the pre-post PVT comparison, I believe the manuscript would benefit from a more cautious interpretation of what this protocol actually measures. At present, the evidence appears to support that the protocol detects changes in cognitive performance following a cognitively demanding task under hypoxia, rather than directly demonstrating cognitive fatigability itself. I therefore encourage the authors to better acknowledge this conceptual distinction and moderate the wording throughout the manuscript, particularly in the Discussion and Conclusions.

2. Ecological validity

The manuscript repeatedly emphasizes the ecological validity and field applicability of the proposed protocol. While I agree that the protocol is portable and considerably more feasible than laboratory-based cognitive assessments, I believe the concept of ecological validity is occasionally overstated. Portability alone does not necessarily imply ecological validity. The cMSIT and PVT remain standardized laboratory cognitive tasks that do not directly reproduce the perceptual, decision-making, or motor-cognitive demands experienced during alpine skiing or other sporting activities. Consequently, I suggest that the authors distinguish more clearly between “field feasibility” and “ecological validity”. Describing the protocol as a portable field-based assessment is fully justified, whereas claiming ecological validity may require a more nuanced discussion and acknowledgement of its limitations.

3. External validity and generalizability

The conclusions occasionally appear broader than the study population allows. The study was conducted exclusively in a relatively small sample of elite youth mogul skiers, yet several statements throughout the Discussion refer more generally to "athletes" or "sport performance." Although the proposed mechanisms may plausibly extend to other athletic populations, the current data do not directly support such broad generalizations. I recommend that the authors explicitly acknowledge that the findings are currently limited to this specific cohort and moderate statements implying wider applicability until further evidence becomes available.

4. Interpretation of practical implications

Finally, I encourage the authors to moderate several statements regarding the practical implications of their findings. For example, the Discussion suggests that "ignoring hypoxia during altitude training could increase injury risk." While this is an interesting and plausible hypothesis, it extends beyond the evidence generated in the present study. The current investigation did not assess injury incidence, technical errors, sport-specific decision-making, or other performance outcomes directly related to injury risk. Therefore, statements of this nature should be presented as potential hypotheses or speculative implications rather than direct conclusions supported by the data. More cautious wording (e.g., "may contribute to..." or "may have implications for...") would more accurately reflect the evidence presented.

**********

7. PLOS authors have the option to publish the peer review history of their article (what does this mean?). If published, this will include your full peer review and any attached files.

If you choose “no”, your identity will remain anonymous but your review may still be made public.

**Do you want your identity to be public for this peer review?**  For information about this choice, including consent withdrawal, please see our Privacy Policy.

Reviewer #1: **Yes:**  WIssem Dhahbi

Reviewer #2: **Yes:**  Ana Filipa Braga Barroso Campos Silva

**********

# Editor report: Acceptance letter

**DOI:** 10.1371/journal.pone.0353673.r006

**Authors:** Julio Alejandro Henriques da Costa, Julio Alejandro Henriques da Costa, Julio Alejandro Henriques da Costa

PONE-D-26-11054R2

PLOS One

Dear Dr. Varesco,

I'm pleased to inform you that your manuscript has been deemed suitable for publication in PLOS One. Congratulations! Your manuscript is now being handed over to our production team.

At this stage, our production department will prepare your paper for publication. This includes ensuring the following:

* All references, tables, and figures are properly cited

* All relevant supporting information is included in the manuscript submission,

* There are no issues that prevent the paper from being properly typeset

You will receive further instructions from the production team, including instructions on how to review your proof when it is ready. Please keep in mind that we are working through a large volume of accepted articles, so please give us a few days to review your paper and let you know the next and final steps.

Lastly, if your institution or institutions have a press office, please let them know about your upcoming paper now to help maximize its impact. If they'll be preparing press materials, please inform our press team within the next 48 hours. Your manuscript will remain under strict press embargo until 2 pm Eastern Time on the date of publication. For more information, please contact onepress@plos.org.

You will receive an invoice from PLOS for your publication fee after your manuscript has reached the completed accept phase. If you receive an email requesting payment before acceptance or for any other service, this may be a phishing scheme. Learn how to identify phishing emails and protect your accounts at https://explore.plos.org/phishing.

If we can help with anything else, please email us at customercare@plos.org.

Thank you for submitting your work to PLOS ONE and supporting open access.

Kind regards,

PLOS ONE Editorial Office Staff

on behalf of

Dr. Julio Alejandro Henriques Castro da Costa

Academic Editor

PLOS One
